# Systematic review of facility-based sexual and reproductive health services for female sex workers in Africa

**DOI:** 10.1186/1744-8603-10-46

**Published:** 2014-06-10

**Authors:** Ashar Dhana, Stanley Luchters, Lizzie Moore, Yves Lafort, Anuradha Roy, Fiona Scorgie, Matthew Chersich

**Affiliations:** 1 Centre for Health Policy, School of Public Health, University of the Witwatersrand, Johannesburg, South Africa; 2 School of Public Health and Preventive Medicine, Monash University, Victoria, Australia; 3 Centre for International Health, Burnet Institute, Melbourne, Victoria, Australia; 4 International Centre for Reproductive Health, Department of Obstetrics and Gynaecology, Ghent University, Ghent, Belgium; 5 School of Public Health, University of the Witwatersrand, Johannesburg, South Africa; 6 MatCH (Maternal, Adolescent and Child Health), Faculty of Health Sciences, University of the Witwatersrand, Durban, South Africa; 7 Ashodaya Samithi, Mysore, India; 8 Wits Reproductive Health and HIV Research Unit, Faculty of Health Sciences, University of Witwatersrand, Johannesburg, South Africa

**Keywords:** Female sex workers, Sub-saharan Africa, Health services, Sexual and reproductive health, HIV prevention

## Abstract

**Background:**

Several biological, behavioural, and structural risk factors place female sex workers (FSWs) at heightened risk of HIV, sexually transmitted infections (STIs), and other adverse sexual and reproductive health (SRH) outcomes. FSW projects in many settings have demonstrated effective ways of altering this risk, improving the health and wellbeing of these women. Yet the optimum delivery model of FSW projects in Africa is unclear. This systematic review describes intervention packages, service-delivery models, and extent of government involvement in these services in Africa.

**Methods:**

On 22 November 2012, we searched Web of Science and MEDLINE, without date restrictions, for studies describing clinical and non-clinical facility-based SRH prevention and care services for FSWs in low- and middle-income countries in Africa. We also identified articles in key non-indexed journals and on websites of international organizations. A single reviewer screened titles and abstracts, and extracted data from articles using standardised tools.

**Results:**

We located 149 articles, which described 54 projects. Most were localised and small-scale; focused on research activities (rather than on large-scale service delivery); operated with little coordination, either nationally or regionally; and had scanty government support (instead a range of international donors generally funded services). Almost all sites only addressed HIV prevention and STIs. Most services distributed male condoms, but only 10% provided female condoms. HIV services mainly encompassed HIV counselling and testing; few offered HIV care and treatment such as CD4 testing or antiretroviral therapy (ART). While STI services were more comprehensive, periodic presumptive treatment was only provided in 11 instances. Services often ignored broader SRH needs such as family planning, cervical cancer screening, and gender-based violence services.

**Conclusions:**

Sex work programmes in Africa have limited coverage and a narrow scope of services and are poorly coordinated with broader HIV and SRH services. To improve FSWs’ health and reduce onward HIV transmission, access to ART needs to be addressed urgently. Nevertheless, HIV prevention should remain the mainstay of services. Service delivery models that integrate broader SRH services and address structural risk factors are much needed. Government-led FSW services of high quality and scale would markedly reduce SRH vulnerabilities of FSWs in Africa.

## Introduction

Female sex workers (FSWs) bear a disproportionate burden of HIV and have high levels of sexual and reproductive health (SRH) morbidity. In sub-Saharan Africa, 37% of FSWs are living with HIV– a figure three times the global HIV prevalence among FSWs [1]. The burden of STIs among this group is also high, with up to two thirds having a curable STI [2]. Several risk factors – such as multiple sex partners, unprotected sex, and unsafe working conditions – place these women at increased risk of HIV and STI acquisition. Interventions targeting these risk factors can substantially reduce risk and consequently infection [3]. Following intervention activities in Cote d’Ivoire, for example, HIV incidence in FSWs declined from 16.3 to 6.5 per 100-person years [4]. After implementation of Thailand’s 100% Condom Use Programme, condom usage among FSWs increased from 14% to 94% [5]. In addition, a modelling study showed that increasing condom use among FSWs from 78% to 90% could prevent up to two thirds of new HIV infections along a trans-Africa highway [6].

Much uncertainty still exists about the ideal service delivery model for FSWs. Should interventions be provided through stand-alone targeted vertical services that only address sex workers and other at-risk populations, or through services integrated within health facilities for the general population [3]? What is the optimum package of health services to provide? A few elements are common to all effective HIV and STI interventions for FSWs: condom promotion and distribution; HIV testing and prevention counselling; and STI screening and management [7]. Yet there may be compelling reasons for expanding this package to include broader health and social services such as cervical cancer screening; family planning and counselling; sexual and gender-based violence prevention and care; as well as interventions that empower and build social cohesion within FSW communities. Structural risk factors, such as discrimination and violence, prevent sex workers from asserting control over their environment and ultimately restrict their access to health services [8]. By creating health-enabling social environments and responding to the broader needs of FSWs, additional services may make other prevention and care programmes more acceptable and accessible to this high-risk group.

Studies among FSWs in Africa have mostly assessed burden of disease, risk behaviours or relative efficacy of individual interventions. Collating information on the design and delivery of services for FSWs in Africa could help to inform future programme design. In this systematic review we examined the models of facility-based SRH-related service delivery for FSWs in Africa; the settings in which these services are delivered; the intervention packages provided; and the extent of involvement of government and international or other donors.

## Methods

On 22 November 2012, Medline (Pubmed interface) and Web of Science were searched, without date restrictions. Within Medline, we combined MeSH and free text terms for low- and middle-income countries [9] together with sex work. In Web of Science, we used text search terms to locate all articles that included Africa or India, and sex work or high-risk populations. We searched the Joint United Nations Programme on HIV/AIDS (UNAIDS) and the World Health Organization (WHO) websites for further reports of sex work programmes, and contacted content experts for additional references. The search included studies in India as this review is nested within a larger comparative study of FSW services in Africa and India.

A single reviewer screened titles and abstracts using EPPI-Reviewer (United Kingdom, version 4) [10]. Duplicate references were removed, and abstracts and titles screened using pre-specified codes. Wherever possible, full text records were assessed for records that did not have an abstract (n = 41) or were marked query by the first reviewer (n = 119). Review methods follow the PRISMA guidelines [11]. Details of outreach and community-based services are described elsewhere.

### Inclusion and exclusion criteria

For the review, a FSW was defined as women who exchanges sex for money or other gifts and commodities. We included any study in Africa (either in sub-Saharan Africa or any other part of the continent) that described facility-based SRH prevention and care services for FSWs. Studies of services solely for other groups at high-risk, such as female bar workers, were excluded, as were studies of services for clients or other partners of FSWs. The review does, however, include studies reporting on services provided for FSWs in addition to other groups, as well as services constructed either within general population services or as targeted stand-alone services only addressing the needs of sex workers and other at-risk populations. Both public- and private-sector services were included. The review aimed to be as inclusive as possible and thus encompassed projects established solely for FSW service provision and sites set up primarily for research purposes, but which nonetheless had extended clinical services to their participants. If non-clinical services were mentioned, data were extracted on these studies, provided they included at least some facility-based SRH services, such as condom provision. We excluded articles that only described population characteristics or needs of FSWs and did not describe interventions for this population. Studies in languages other than English were excluded, as were studies based solely on mathematical modelling of interventions.

### Study variables and analysis

**Figure 1:**
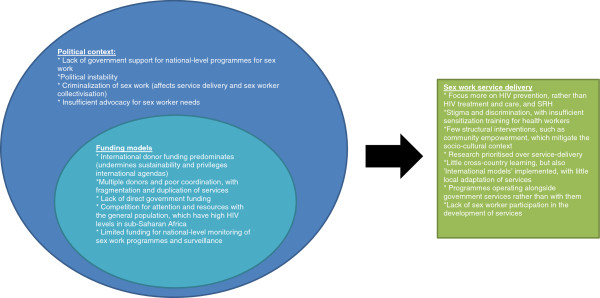
Conceptual framework depicting why sex worker interventions have not scaled-up in Africa.

A single reviewer extracted data from included articles using standardised tools. Variables extracted encompassed general characteristics of the services provided (year started, country, and part of city where services are provided) and service settings (facility and its setting, types of buildings, or use of mobile clinics). Service delivery models were classified as stand-alone services, or whether all or any components were integrated with general population services. Data were extracted on the clinical and social services provided in each project. Finally, we extracted – from the main article as well as its acknowledgments section and footnotes – any mention of support or involvement of government, organizations, or other donors in each project.Key themes across the studies are summed along with novel service delivery examples that illustrate exceptions to common themes. Analysis was mostly qualitative, though the proportions of projects with specific characteristics are also presented. Where possible, lessons are then drawn for future design of FSW interventions in these settings. Finally, we collated all information to form a conceptual framework depicting the interacting factors which likely determine the service delivery models and their coverage (Figure 1).

## Results

**Figure 2:**
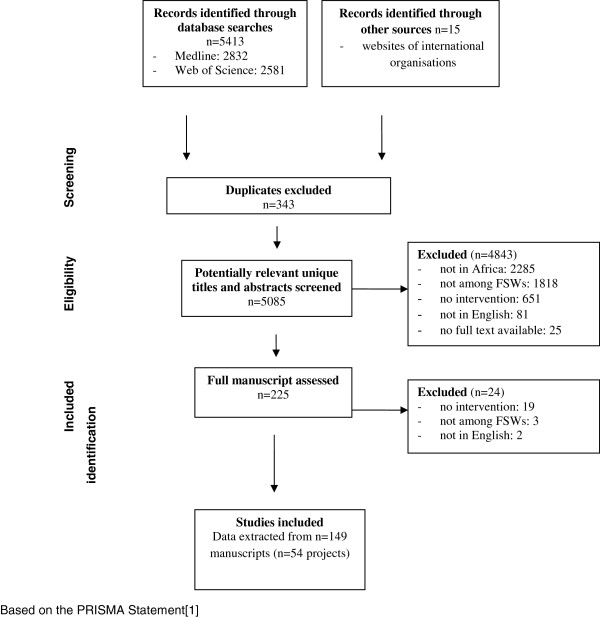
**Flow diagram of studies selected for systematic review of sex work services in Africa.** Based on the PRISMA Statement.

We located 5413 articles (Figure 2) of which 343 were duplicate references, 2159 reported on studies conducted in countries other than Africa, and 1818 were among population groups other than FSWs. A further 81 non-English language studies were excluded as were 651 studies among FSWs that did not describe a relevant intervention. In total, we included 149 articles describing 54 projects in Africa.

### Models of service delivery

Of the 47 projects that document the year in which services began, 19 began in the period after 2000, while 15 began between 1990 and 1999, and 13 in the 1980s (Additional file 1: Table S1). We located services specifically for FSWs (either targeted services for sex workers and other at-risk groups, or those integrated within general services) in only 28 of the 58 countries located in Africa. Much of the data we identified reported on sites that provided services as part of research projects among this population, rather than on sites that focused exclusively on service delivery.

Services were commonly situated in sex work hot spots within national or regional capital cities of a country. Other settings included truck stops, borders, ports, and mining areas. Of note, two sites in Zimbabwe reported specifically providing rural-based services, either on farms or near mines [12,13]. Of the 54 projects, 15 involved some form of outreach, either to streets, bars or other places of entertainment, and rarely to brothels [14]. Outreach was provided either through the use of mobile clinics, or through peer workers or counsellors. Visits to FSWs’ homes were also reported in Benin and Kenya [15,16].

Most projects were localised and small-scale and were often confined to a single sex work hot spot; only about two thirds provided services at more than one point or city in a country, and few projects extended over large geographical areas and populations. On the whole, explicit networking or cross-learning between FSW projects was uncommon. A noteworthy exception was the establishment of services in Kisumu Kenya in 2006, which were modelled on the Abidjan Cote d’Ivoire project, operational since 1992 [17,18]. In general, most services developed from the ground up and not as part of a national coordinated approach. In Kenya and Ghana, however, there were examples of services that began as single clinical sites and then expanded to other areas [19-23].

We did, however, find some initiatives to provide regional coordination of projects. The West African Project to Combat AIDS and STIs provided preventive and curative care to FSWs and their clients in nine West African countries, with funding from the Canadian International Development Agency [24]. Coordination of services along several major transport routes was also noted [25-29]. In the West Africa Highways Project, over 36 public, private and community health facilities were upgraded, equipped, and provided with an adequate supply of drugs in five countries. One national level project was also attempted in Malawi, but appears to have reached low coverage overall [19].

Most sites specifically targeted FSWs, but a few also offered services to key client groups such as truckers, miners and even military personnel. Services for client groups, however, were mostly provided through outreach, rather than by specialised clinics. Of note, two sites in Namibia and Kenya also offered services for other high-risk groups such as men who have sex with men (MSM) [30,31]. Most FSW services along major highways were integrated with services for other high-risk groups.

Night-time or after-hours services were mentioned in only four reports. No clinic offered 24 hour services, but one clinic in Benin was open up to 01:00am [16]. Frequency of contact was variable, ranging from weekly to every four months, but most projects aimed to have contact with FSWs either monthly or two-monthly.

Overall, specialized facility-based services for FSWs were prominent, with few opting to integrate services within existing facilities. In a study of targeted services for FSWs and truck drivers in Tete, Mozambique, all the interviewed informants endorsed the notion of separate SRH clinics and the need to expand these services to other areas in the country. FSWs reported high satisfaction with the clinic, mentioning good reception by staff, prompt attendance, close proximity and free health services as measures of satisfaction. Women, however, wanted the range of services and opening times to be expanded [32].

### Package of clinical services in African FSW projects

Virtually all projects focused on HIV and STI interventions rather than on broader SRH services. Condoms were provided as part of almost all clinical services as well as within peer outreach or sex work settings (Additional 1: Table S2). In some instances bar owners assisted with condom distribution [33]. Provision of automated condom dispensers appeared rare. Free condom distribution was common, as was condom promotion and distribution within a social marketing or social campaign approach [28,34-40]. Female condoms were distributed by less than a tenth of all services. In Madagascar, staff advised FSWs to use female condoms only when male condoms could not be used [36]. One site in Zimbabwe provided instruction sheets on female condom use, and women practiced insertion in the presence of a trained project nurse [41]. Another site recruited FSWs to study the safety, acceptability and feasibility of female condom re-use and lubrication [42]. Overall, however, water-based lubricant, for use with either male or female condoms, was provided by only three projects [31,39,42,43]. Condom promotion, often led by peer workers, included demonstrations and training on negotiation skills, as well as the correct storage, use, and disposal of condoms. While condom-use negotiation skills may have been a part of condom promotion activities, they were only explicitly mentioned in nine projects. Education on condom use was either clinic or community based. Notably, innovative approaches to education activities were employed by projects in Cote d’Ivoire and along the West African highways; these activities included promoting condom use through picture albums, videos, cue cards, drawings, billboards, radio, calendars, t-shirts, and commercials. Advertisements in these projects were localized to reduce project costs [24,44]. In Malawi, nightclub disk jockeys promoted condom use and distributed safe sex messages [45]. Only a few sites encouraged an explicit, standardised “no condom no sex” approach [36,46]. By contrast, a site in Pretoria, South Africa, used a personalised approach to assess each FSWs risks and goals for negotiating risk-reduction [47].

HIV testing and counselling (HCT) was common (35/54), including testing for HIV-2 in many West African sites. HCT was mostly done at clinical centres, but also through mobile clinics and peer outreach. Repeat HCT for HIV-negative women was less common and offered either monthly or 3-monthly, while a few sites tested 6 or 12 monthly. No site reported offering HCT to couples or partners. Provision of HIV care – CD4 cell count testing (7/54), cotrimoxazole prophylaxis (3/54), antiretroviral therapy (ART) (3/54), and prevention of mother-to-child transmission (PMTCT) (1/54) – was mentioned by few services, with the majority referring HIV-infected women to other centres for HIV care and treatment. In the West African Highways project, free ART was provided to over 500 people, but treatment was mainly offered to vehicle drivers who tested positive. A referral system was, however, established for those eligible for ART [24]. ART services in Burkina Faso did include adherence support and active tracing of women who defaulted follow-up [48]. In that country and in Kampala, Uganda, women received pre-ART counselling and, to reduce potential discrimination, a staff member would then physically accompany them to the local ART clinic [48,49]. In Namibia, health workers underwent training and sensitization to reduce stigma and improve care and support for FSWs [37]. By contrast, concerns around confidentiality meant that some FSWs at a site in Rwanda elected to obtain ART at more distant sites. Oral pre-exposure prophylaxis against HIV was only offered in Kilifi, Kenya [31]. No site specifically mentioned providing post-exposure prophylaxis following either unprotected sex or sexual abuse. There was also no specific mention of care and treatment services for other HIV-related comorbidities such as tuberculosis.

Most STI interventions encompassed STI education, screening, and syndromic or aetiological management. History and pelvic examination – often with a speculum, and to a lesser extent, by colposcopy – was common, even for asymptomatic women. Screening tests often included visualization of the cervix; collection of vaginal and cervical samples; and other laboratory tests. Screening for syphilis, trichomoniasis (TV), gonorrhoea (NG) and chlamydial infection (CT) was common, though testing for candidiasis (CA) and bacterial vaginosis (BV) was less frequent. Testing for herpes simplex virus type 2 (HSV-2), M.genitalium and H.ducreyi was uncommon. At the Majengo clinic in Nairobi, expansion of existing infrastructure made polymerize chain reaction (PCR) testing for STIs possible [21]. Intervals between STI screening visits were mostly monthly or three monthly, and even weekly in one instance in Malawi [50]. Some sites in Madagascar offered different STI services at first visits compared to later visits, which were risk-score based [51,52]. Similarly, women new to the clinic’s services initially had visits scheduled at closer intervals, but then over longer periods over time. A novel approach to providing STI care was offered in Malawi, where services were offered at places of entertainment [45]. Partner tracing and notification was rare and only mentioned by five sites. Peer-led education sessions on STIs, however, were common. Most sites reported using WHO or national STI screening and syndromic management algorithms. Use of risk assessments to guide STI treatment was used at two sites [28,53]. Acyclovir treatment for HSV-2 may have been included in management guidelines, but was only specifically mentioned in a few instances. No services offered immunization or testing for hepatitis B. In contrast to frequent STI screening services, we located only 11 reports of periodic presumptive treatment (PPT), which consisted of azithromycin alone or in combination with ciprofloxacin, doxycycline, or metronidazole. Regimens aimed to cover CT and NG, though PPT for CA, TV and BV was also used. Treatment intervals varied, from single-dose therapy to once every one to three months.

In contrast to STI and HIV services, few sites reported providing general or primary health care services; even fewer offered broader SRH services. We located only six instances in which urine pregnancy testing was offered. Contraceptive services were similarly only available at seven sites: the East and Central African Highways project, Kenya, Madagascar, Malawi, Mozambique, South Africa, and Uganda. Few projects specifically promoted dual methods of contraception. Unlike at other sites, services in Madagascar covered a range of contraception methods, offering emergency contraception and the diaphragm [54]. The latter was also provided in a study in Mombasa, Kenya [55]. No programme mentioned termination of pregnancy services. Interestingly, a general care clinic in Uganda offered health services to children of FSWs [49]. Overall, access to cervical cancer screening and treatment (with cervical smear or colposcopy) was only reported in Senegal, South Africa, and Gambia [56-58]. Two sites, in South Africa and in Kenya, tested for Human Papilloma Virus (HPV) subtypes [59,60], but there were no instances in which services offered vaccination to prevent HPV.

Finally, few programmes provided structural interventions to address environmental sources of vulnerability and ill health. Gender-based violence services were only offered in Zambia and South Africa. In Zambia, legal protection was arranged through referrals from the local clinic, while FSWs were sent to government hospitals for violence-related health needs [61]. Only one project in Cape Town, South Africa, provided legal literacy for FSWs [62]. To reduce risk and achieve more independence, FSWs at another site in South Africa learnt violence prevention strategies and methods, such as developing concrete personal plans to reduce risk. In this programme, each woman’s drug and sexual risks were also assessed [47,63]. No other sites were identified providing harm-reduction services for FSWs who inject drugs or consume alcohol. In Kenya, an income-generating project provided micro-enterprise services to FSWs [64], whereas a project in South Africa made an effort to improve literacy through educational programmes [56]. Another income-generating project that helped FSWs develop tailoring businesses was identified in Malawi, but reportedly only six FSWs were assisted [45].

### Government involvement and key funders of services

We located no services for FSW that were fully government-funded; most were supported by a range of international donors. Although some involvement or approval from local authorities was reported by about a third of projects, direct government funding, or provision of FSW services within public-sector clinics, was largely absent.

Of 49 projects that documented either a funder or an organization responsible for the project, 31 (63%) had reported funding from at least one North American organization – Canada and/or United States of America (USA) - and 20 (41%) had at least one European donor. The President’s Emergency Plan for AIDS Relief (PEPFAR) or the United States Agency for International Development (13/49), as well as Family Health International (12/49), predominated among the USA funders, and many projects centred on research received funding from the USA Centre for Disease Control and Prevention (4/49) and the National Institutes of Health (4/49). European funders included the European Commission as well as individual European countries. Few private funding sources (commercial or donors) were acknowledged. We did, however, locate two instances of mining companies involved in sex work projects, both in Southern Africa [13,65].

A sex worker clinic in Tete, Mozambique, reported novel arrangements between the government and external funders [32]. The government partly funded capital and operational costs, and provided health staff and medical supplies, while external funders contributed to other costs such as medical equipment and furniture, utilities, three security guards, over-time for nurses, training and educational materials, and peer educator costs.

User fees on the whole were uncommon. Some sites charged FSWs for commodities such as condoms or emergency contraception [22,57,66]. In some projects, only a pre-defined number of condoms were given to FSWs at no cost [16]. A site in Nigeria sold condoms at subsidized prices, while emphasising that they helped to save FSWs money on health services such as antibiotics for STI treatment. There were, however, two sites in Ghana and Guinea that employed a cost-recovery model for the management of health problems other than STIs [57]. In a few instances, the donor-funded model clearly limited sustainability, with interruptions in international funding leading to some services being discontinued. In projects in Ghana and Kenya, for example, condoms could no longer be provided for free, and other services such as STI diagnosis and treatment and HIV testing had ceased [67,68]. FSWs in Madagascar were given sufficient condom supplies for only a few years of the project. For the years when funding was limited, women received some free condoms and were advised to purchase additional ones from either peer educators or social-marketing agents [69,70].

## Discussion

Overall, this review found that sex work programmes in Africa are localised and small-scale; operate with little coordination, either nationally or regionally; and have scanty government support. While most programmes provide several HIV prevention and STI interventions, few address sex workers’ broader reproductive health or ART needs, or the underlying structural drivers of vulnerability and risk. Furthermore, many projects are couched within research activities; few concentrate on ongoing, large-scale service delivery. More broadly, HIV prevention efforts in Africa have successfully reduced HIV transmission to children, and among the general population through interventions such as HCT and medical male circumcision [71]. But compared with FSW projects in several countries, those in Africa have given much less systematic attention to altering risk for HIV in FSWs, their clients and emotional partners.

Few countries in Africa have systematic means of monitoring coverage and outcomes of programmes for sex work. In the 2012 UNAIDS global report, only 13 provided data on coverage of HIV prevention programmes for sex workers [72]. Of these, five had coverage under 50% and a further four reported access levels of 50-74%. Coverage is likely even lower in countries that did not provide data and similarly in the 30 countries where we did not locate publications of services for FSWs. In Ghana, Accra, which has an estimated 5000 sex workers [73], a project was able to provide STI treatment to only 296 FSWs who lived in the city [74], while in Yaoundé, Cameroon, which has a similar sized sex worker population, only 303 sex workers were offered female-controlled methods to protect against STIs [75]. Through a peer-mediated intervention in Mombasa, Kenya, 62 peer educators were able to reach about a third of Mombasa’s sex worker population [20]. By contrast, in Cotonou, Benin, close to 100% of FSWs knew of the project’s dedicated sex worker clinic and 81% had previously attended the clinic [16].

Though most African countries address sex work in their multisectoral AIDS strategies, few governments provide direct funding for national-level programmes for sex workers. Recently, internationally funding for sex work services has risen, following many years of stagnation, which was at least in part due to PEFAR’s “Anti-prostitution Loyalty Oath” [76]. Globally, among 30 countries that reported spending for sex worker programming (with data available for at least one year in 2006–2007, 2008–2009 or 2010–2011), total spending rose 3.7-fold during 2006–2011. Nevertheless, international funding for sex work mostly focuses on HIV prevention [72]. For example, from 2002 to 2009, The Global Fund to Fight AIDS, Tuberculosis and Malaria only allocated about 0.5% of its funding towards MSM, sex workers, and injecting drug users. Only 4% of this figure (0.02%) was designated for treatment – the remainder was assigned for HIV prevention [77]. Funding patterns challenge the sustainability of programmes for sex workers; international sources account for much of recent funding increases, constituting an estimated 91% of total spending on HIV programmes for sex workers in 2010–2011. Poor sustainability of projects has important adverse health consequences. Aside from the need for consistent supplies of commodities such as condoms [78], regular long-run contact between providers and sex workers is needed for sustained behaviour change and PPT for STIs, for example. Of greater concern, limited sustainability may undermine trust and confidence in health services. Where access to health services is poor, many FSWs use traditional healers or purchase over-the-counter drugs [79].

In addition to funding constraints, several other factors may undermine coverage of FSW services in Africa (Figure 1). Criminalisation of sex work hinders service delivery and sex worker collectivisation, and raises HIV and other risks for this population [80]. Also, compared with many other settings, sex work in Africa is hard to distinguish from transactional sex, which is very common in this setting. Few reports were located of well-circumscribed sex work settings, such as brothels, which might make programming easier. Further, many countries in the region have alarmingly high levels of HIV in the general population, and thus sex work programmes, quite rightly, compete for resources and attention with general population initiatives.

Scaling up HIV prevention interventions among FSWs and other high-risk groups appears cost-effective, and costs reduce with increases in coverage [81]. Combining multifaceted approaches may also have synergistic rather than additive effects. Women in a South African mining community, for example, said that they were tired of receiving condom messages while other health issues were overlooked. Once STI services were introduced – in response to their expressed needs - condom use increased [82]. In general, however, the African projects reviewed here tend to incur high costs of international staff and operate without economies of scale. The authors of a study in Tete, Mozambique, argued that the average running costs of a dedicated FSW clinic were comparable to the costs of integrating services for high-risk groups within general population facilities. Increasing the number of sex workers attending the clinic would lower the cost per visit considerably [32].

Some individual projects detailed here had evolved over time, with modifications in the package of services provided and their delivery platforms. Yet, no overall pattern of progressive maturity or stages of development were discernable across African sites. In other settings such as India, two distinct patterns characterised service provision. Firstly, the uncoordinated and mostly small-scale services analogous to current sex work models in Africa, followed by large-scale, more uniform projects operational since 2003 [83]. The Avahan India AIDS Initiative shows that effective programming requires coordinated action across several actors and programme levels. Large international donors played a key role in establishing state-wide FSW services, but the Indian government then assumed responsibility for these projects, integrating them within larger national programmes. Avahan works either as the sole service provider in a district or alongside government or donor-supported nongovernmental organizations (NGOs) [84]. The programme includes state-level partners that contract local NGOs. These NGOs then organise clinical services, peer outreach and community mobilisation. By 2009, coverage of these services reached almost 80% [85].

Several large projects were located along major transport routes in all regions of Africa. Aside from scale, these projects had key similarities with other sex work projects on the continent, in that they tend to operate in parallel with government services and have difficulty sustaining their services. In contrast, the Indian highway project fell within the Avahan programme, which was then responsible for coordinating services across the transport routes of five states. Though some local adaption is required, coordination of projects allows for sharing of effective approaches and for advocacy at regional and national levels. On the other hand, poor coordination and multiple donors often leads to fragmentation and duplication of services [2]. Moreover, sex workers frequently move in search of work, or for other reasons such as political instability, and are often not citizens of the countries in which they live – a further motivation for cross-border collaboration [86].

Importantly, some countries in Africa did integrate sex worker programmes within existing health facilities [8,34]. Separate services for FSWs may counter fears of discrimination and rapidly enhance access to specific SRH services, but they can also inadvertently displace these women from more integrated health services, where FSWs may have access to key services such as ART [87]. FSW-only facilities perpetuate the notion that general-population services are inherently discriminatory, reinforcing fears of accessing healthcare within an integrated system. For instance, once a separate STI clinic in the Democratic Republic of Congo was opened to the general public, FSWs attendance at the clinic dropped substantially [88]. Likewise, separate STI services along a highway in Tanzania had a higher utilization of services than integrated services along the same route [28].

Several key SRH needs of FSWs appear unmet. Rates of unwanted pregnancies are high in this population. In three African countries, 35-86% of FSWs had at least one previous abortion [89-91]. Unintended pregnancy contributes to economic difficulties and an inability to exit sex work and refuse a client unwilling to have protected sex [23,55]. FSWs in South Africa, for example, cited financial support for their dependent children as the main reason for engaging in sex work [92]. In four Madagascan cities, nearly 30% of FSWs stated that preventing pregnancy was moderately to very important [90]. Poor access to family planning services means many FSWs resort to less reliable contraceptive methods, such as condom use alone [90,93]. Female-initiated barrier methods appear under-utilised, although they are acceptable, provide added protection, and result in significant declines in STI prevalence among sex workers [36,94-96]. Also, screening for cervical cancer is rare among FSWs – a group with a higher prevalence of abnormal cervical cytology than the general population [40,97]. As well as offering cervical cancer screening, further efforts are needed to improve access to the HPV vaccine [98]. Supported by findings of a systematic review and modelling studies, WHO recommends that PPT be offered when other approaches are impractical, where clinical services are limited, or as part of a comprehensive STI service package [7,99,100]. PPT as a treatment approach, however, is underutilized in Africa.

Presently, few sites actively trace FSWs who fail to return for STI results or ensure that those referred for ART initiate therapy. Rapid point-of care (POC) tests may eliminate the need for follow up, allowing for diagnosis and treatment of STIs in one visit [101]. In addition, up to 60% of FSWs in Africa are unaware of their HIV status, and almost half of HIV-infected FSWs in Ghana discovered their status during pregnancy [23,102]. Thus, family planning services may play a key role in mitigating PMTCT. Adequate access to HCT facilitates access to care and support services, though there is little evidence of behaviour change following HCT among sex workers [3]. Given high HIV incidence, re-testing warrants additional thought within future FSW programmes. WHO recommends that adults aim to retest six weeks after a possible exposure and then 6-monthly [103].

Disappointingly, FSW services seemed to place little emphasis on HIV treatment and care, despite marked gains in ART access globally [104]. While it is true that ART access, for all populations, was limited for much of the period under review, it is concerning that we did not locate evidence that access had increased in recent years. Inadequate sensitization training, discriminatory laws, and the dual stigma from HIV and sex work likely further hinder FSWs access to HIV treatment and care [3,105]. ART is central to securing the health of sex workers and preventing onward transmission. This must be a key part of future sex work programming and could have a marked impact. Over a 5-year period in San Francisco, for instance, a citywide effort to test and actively link MSM to treatment was associated with a 40% decline in community viral load and a 45% reduction in new infections [106].

The environments in which sex work generally occurs limit the ability of FSWs to control their individual health risks. In Africa, many FSWs experience some form of violence – either in their personal or professional lives [92,107]. Besides its impact on emotional wellbeing, sexual violence heightens risk of unintended pregnancy, gynaecological morbidity, STIs, and HIV [108-110]. Multi-level interventions elsewhere have successfully reduced violence among sex workers. These programmes directly tackled the politico-legal context, mobilized the FSW community, and provided services such as 24 hour crisis management teams, medical care (including post-exposure prophylaxis), counselling, and legal support [83,111-113]. Aside from a few examples, such as micro-enterprise services in Kenya [64], few interventions in Africa appear to address the structural risk factors which hinder FSWs ability to access health services [113,114] and protect their wellbeing and health [8,115].

This review has several limitations. Only projects in Africa were examined; thus, our findings may not apply to other settings or to the sizable population involved in transactional sex in Africa. Generalizability across Africa may also be limited by socio-cultural variations between settings. Also, the paper focused specifically on describing the composition of interventions delivered, not their uptake, effectiveness or cost-effectiveness. Further, we may have raised the likelihood of errors, as screening for eligibility and data extraction was not done in duplicate. Moreover, and most importantly, we did not review grey literature and non-peer reviewed journals, especially those which may document structural interventions such as Research for Sex Work [116], and we likely also missed many effective projects run by non-governmental organisations which remain unevaluated. Doubtless, such projects would have provided much useful information for this review.

## Conclusions

We found marked limitations in the service delivery models used by African FSW projects, which have low coverage, are fragmented and donor-led, and seldom benefit from economies of scale. It is essential that governments in Africa increase their support for sex worker initiatives, and move to integrate them into larger and more sustainable national programmes. While HIV and STI prevention remains the mainstay of services, access to ART - which is important not only for securing the health of FSWs, but also for reducing further HIV transmission - needs to be addressed urgently [117]. Prevention of transmission to emotional partners, with whom consistent condom use is the lowest, remains a crucial issue [118]. Rapid POC tests improve the diagnostic accuracy of STI screening and counter the high rates of loss to follow up among FSWs, while PPT – as a STI treatment approach – is simple and cost effective to implement in these settings. Moreover, service delivery models that are responsive to the expressed wider health and social needs of sex workers are required, especially those that provide broader SRH services such as cervical cancer screening, dual family planning methods, and gender-based violence services. Structural interventions, such as empowering sex workers and decriminalizing sex work, have the potential to foster an environment conducive to safer sex and service delivery. Much more effort is needed to make the work of FSWs safer in Africa, and the public health and human rights imperative to achieve that should take precedence over moral or legal concerns of societies or funders. Government-led FSW services of adequate quality and scale would markedly reduce SRH and HIV vulnerabilities of sex workers in Africa.

## Abbreviations

FSW: Female sex worker; HIV: Human immunodeficiency virus; STI: Sexually transmitted infection; SRH: Sexual and reproductive health; ART: Antiretroviral therapy; USA: United States of America; UNAIDS: Joint United Nations Programme on HIV/AIDS; WHO: World Health Organization; MSM: Men who have sex with men; HCT: HIV testing and counselling; PMTCT: Prevention of mother-to-child transmission; PPT: Periodic presumptive treatment; TV: Trichomoniasis virginals; NG: Neisseria gonorrhoea; CT: Chlamydia trachomatis; CA: Candida albicans; BV: Bacterial vaginosis; HSV-2: Herpes simplex virus type 1; PCR: Polymerize chain reaction; HPV: Human papilloma virus; NGO: Nongovernmental organization; POC: Point-of care; Pap: Papanicolaou; PHC: Primary Health Care.

## Competing interests

The authors declare that they have no competing interests.

## Authors’ contributions

All authors made a substantial contribution to the conception and design of the review and have been involved in drafting the manuscript or revising it critically for important intellectual content. AD drafted the manuscript. All authors read and approved the final manuscript. AD, MFC and LM reviewed original studies and extracted data from eligible articles. SL supervised the analysis and interpretation of the data.

## Supplementary Material

Additional file 1: Table S1. Target groups and service access in African sex work projects. **Table S2.** Package of clinical services provided in African sex worker projects. Click here for file
