# Supplementary material for: Systematic review of facility-based sexual and reproductive health services for female sex workers in Africa
Source: Global Health. 2014 Jun 10;10:46. doi: 10.1186/1744-8603-10-46 (PMC4070634; doi:10.1186/1744-8603-10-46)
Supplement: Additional file 1: Table S1. — Target groups and service access in African sex work projects. Table S2. Package of clinical services provided in African sex worker projects. [file 1744-8603-10-46-S1.docx]

**Supplementary Table 1: Target groups and service access in African sex work projects**

|  | **Country, City** | **Year began** | **Service delivery model (stand-alone, mixed/diagonal, integrated; and whether any components were integrated with general population services)** | **FWS population targeted, facility and setting** | **Time and days clinic services available** |
| --- | --- | --- | --- | --- | --- |
| 1 | Benin, Cotonou, Porto Novo, 10 small town clinics across country [[1-13](#_ENREF_1)] | 1992 | A dedicated clinic and visits at home or in the workplace by fieldworkers. | Intervention targets FSW and their male partners. 1 specialized clinic in Cotonou, 2 in Porto Novo and 10 other small town clinics) | Monthly visits, either in field or clinic. Field workers approach male clients 8pm to 1am, just prior to going into room for sex with FSW. |
| 2 | Burkina Faso, Bobo-Dioulasso [[9](#_ENREF_9), [14-18](#_ENREF_14)] | 1998 | Dedicated clinic within a public health facility | Open public facility for medical consultation, environmental hygiene and vaccinations | Follow-up visits every 3 or 4 months. |
| 3 | Cameroon, Yaounde and Douala [[19-22](#_ENREF_19)] | 1989 | Dedicated study clinic | Monthly clinic visits scheduled. | Monthly follow up. Clinic opening times not described. |
| 4 | Cote d' Ivoire, Abidjan, and 11 other towns [[23-33](#_ENREF_23)] | 1992 | Dedicated FSW clinics. Eleven primary health care (PHC) clinics with an integrated package of services for sex workers. Clinic is also a research centre. | 2 Confidential clinics (special FSW clinics), in a non-stigmatizing and confidential environment. | Normal working day |
| 5 | Democratic Republic of Congo, Kinshasa, Matonge[[34](#_ENREF_34)] | 1985 | A dedicated women's health centre, FSW and STI clinic | Dedicated FSW and STI clinic | Not stated |
| 6 | East and Central African highways [[35](#_ENREF_35)] | 2005 | Special outreach services including recreation and resource centres, with educational outreach for high-risk groups | Transport corridors in Burundi, Democratic Republic of Congo, Djibouti, Ethiopia, Kenya, Rwanda, Sudan, Tanzania and Uganda. | Not stated |
| 7 | Ghana, Accra [[36](#_ENREF_36)] | Not stated | Community-based outreach intervention for FSWs | Community-based services, including in homes of FSWs and pimps | Not stated |
| 8 | Ghana, Accra, Kumasi, Techiman [[5](#_ENREF_5), [37-42](#_ENREF_37)] | 1987 | Dedicated clinics for FSWs, Ghana Red Cross drop-in centres. | Initially Accra, extended to clinics in 3 cities | Monthly visits, in field or at clinic |
| 9 | Guinea, Conakry [[43](#_ENREF_43)] | Not stated | FSW services integrated into antenatal  clinics or general health care | Centre de Sante´ de Madina, CHU de Donka | Monthly visits, in field or at clinic |
| 10 | Kenya, Kisumu [[44](#_ENREF_44)] | 2006 | Integrated clinic with free services. | Targeting FSWs. Intervention modelled after clinic in Cote d’Ivoire | Not stated |
| 11 | Kenya, Mombasa, Kisauni [[45-49](#_ENREF_45)] | 2000 | Peer workers conducted 1-on-1 or weekly-group sessions, mostly in FSWs houses or at a drop-in centre within community | FSWs in target area, both clinics in areas with high FSW population and community outreach services | Not stated |
| 12 | Kenya, Mombasa, Ganjoni [[50-59](#_ENREF_50)] | 1993 | Ganjoni Municipal STI clinic. | Open-cohort study, monthly visits. Targeting FSWs | Not stated |
| 13 | Kenya, Nairobi, Mukuru [[60](#_ENREF_60)] | 2003 | Local Mukuru community health clinic | FSWs and male clients | Not stated |
| 14 | Kenya, Nairobi, Majengo Observational Cohort Study (MOCS) [[61](#_ENREF_61)] | Not stated | Dedicated FSW clinic | Free comprehensive health care | Not stated |
| 15 | Kenya, Coast Province, Kilifi District, Mtwapa [[62](#_ENREF_62)] | 1984 | Comprehensive HIV prevention package to at-risk research volunteers | MSM and FSWs. Two cities in Kenya, Nairobi (the capital city) and Mtwapa (a mid-size coastal town) in Kilifi District | Not Stated |
| 16 | Kenya, Nairobi, Kibera [[63-68](#_ENREF_63)] | 1985 | Nairobi research clinic (PHC model). Community outreach services provided. | FSWs were enrolled in a clinical  trial | Not stated |
| 17 | Kenya, Nairobi, Pumwan, Majengo[[69-73](#_ENREF_69)] | 1985 | PHC clinic and community outreach programme. | FSWs cohort. Expanded to include other slum area and 3 other outreach towns | Not stated |
| 18 | Kenya, trans-Africa highway[[74](#_ENREF_74)] | Not stated | Outreach services along highway stops. | 3 stops along highway, with 1000, 2000, 5000 inhabitants. FSWs, truck drivers and their assistants, young men living and working at truck stops | Not stated |
| 19 | Madagascar, Antananarivo, Tamatave, Antsiranana, Mahajanga, Toamasina[[75-86](#_ENREF_75)] | 2000 | Non-government facility providing community-based education and clinical services to STI patients and FSW (''67 Ha Clinic''). Counselling also provided in the community. | Public dispensary. FSW | Visits scheduled 2-3 monthly, monthly in 1 study |
| 20 | Madagascar, Diego-Suarez[[87](#_ENREF_87)] | 2000 | Public STI clinic | FSWs | Plans to increase opening hours of clinic |
| 21 | Malawi, 3 towns in Thylo District (Thyolo, Luchenza and Bvumbe)[[88](#_ENREF_88), [89](#_ENREF_89)] | 1999 | FSW-services provided via mobile clinic at designated rest house facility | FSWs registered at bars and rest houses as beer-servers/cleaners/food handlers. Room available for medical examinations and confidentiality of data ensured | Once a week during daylight hours. Weekly follow-up visits |
| 22 | Malawi, Dowa and Lilongwe Districts [[90](#_ENREF_90)] | 1990 | Peer services provided in bars and trucking companies | 3 districts towns adjacent to capital city (Lilongwe) and on main trucking routes from Tanzania and Zambia. Program expanded nationwide. Peer services provided in bars | Not stated |
| 23 | Malawi[[91](#_ENREF_91)] | 2004 | Peer services in places of entertainment (e.g. disco houses, pubs) | Sex workers and owners, disk jockeys | Not stated |
| 24 | Mali, Bamako[[43](#_ENREF_43)] | 2000 | Clinic, uncertain if only for FSWs | Clinic called Centre de Sante´ d’Asacobafa and Commune-6 | Weekly follow-up |
| 25 | Mozambique, Tete, Moatize[[92](#_ENREF_92)] | 2001 | Public sector run dedicated clinic. All services free of charge | Targets SWs and truck drivers. Clinic is 2 converted shipping containers near major truck stop ("Tete Corridor" connecting Zimbabwe with Malawi) on outskirts of Moatize town, 20km from Tete | Mon-Fri, 16:00-22:00pm (time considered convenient by target populations) |
| 26 | Namibia, Oshakati[[93](#_ENREF_93)] | 2000 | Peer services at larger centres along the major thoroughfares, and border towns/ports | High-risk groups' such as female sex workers | Weekly behaviour change communication sessions held by peer educators. |
| 27 | Namibia, Katima Mulilo, Oshikango, Windhoek, Keetmanshoop, Walvis Bay and Swakopmund [[94](#_ENREF_94)] | Not stated | Dedicated clinics and integrated services | Most at risk populations: MSM; FSWs and clients, i.e. truckers, seafarers and miners | Not stated |
| 28 | Nigeria, Asawara state[[95](#_ENREF_95)] | 2006 | Dedicated research clinic for FSWs | Targeting FSWs 18-35 years living in brothels | Not stated |
| 29 | Nigeria, Cross River State, Calabar and Ikom. Also Port Harcourt, Rivers State[[96](#_ENREF_96)] | 1989 | Mixed model (services for FSWs in an STI clinic) | STI clinic mainly, also other health and preventative services | Follow up 6 monthly/yearly. Separate clinic hours for clients. Clinic hours revised to suit needs of FSW |
| 30 | Nigeria, Jos[[97](#_ENREF_97)] | 2006 | A private health facility was used as the study site. The facility also provided services for the general population | Brothel-based FSWs living in urban Jos-Bukuru environs | Not stated |
| 31 | Rwanda, Kigali[[98-101](#_ENREF_98)] | 2006 | Dedicated FSW clinic | Research NGO, with research clinic and laboratory | Not stated |
| 32 | Senegal, Dakar [[102](#_ENREF_102)] | 1970s | National specialist medical centres for FSWs | FSWs required to register with 1 of 4 national specialist medical centres for FSWs (IHS is Dakar Centre) | Not stated |
| 33 | Senegal, Dakar, Kaolack, Ziguinchor and Saint-Louis [[102](#_ENREF_102)] | 1986 | Management of STDs is integrated into regular primary health care services | Services targeting FSWs began after National AIDS Prevention Committee in 1986 to respond to HIV epidemic. Most services since then | Not stated |
| 34 | Sierra Leone, Port Loko[[103](#_ENREF_103)] | 2000 | Activities specifically target sex workers and military personnel. Education and outreach efforts among these groups | Specifically targeting FSWs and military personnel. | Not stated |
| 35 | South Africa, Johannesburg, Hillbrow[[12](#_ENREF_12), [104-108](#_ENREF_104)] | 1996 | Dedicated clinic. . Mobile and permanent clinic. | Targeting SWs | Mobile clinic 3 to 4 days/month, introduced into 12 of 25 hotels. Clinic operated on Thursday evenings |
| 36 | South Africa, Cape Town [[109](#_ENREF_109)] | 1996 | Dedicated services | Based at SW NGO offices (Sex Worker Education and Advocacy Taskforce, SWEAT) | Not stated |
| 37 | South Africa, Carletonville[[110](#_ENREF_110)] | 1998 | Dedicated clinic | Community based intervention to control HIV and STI spread. 2 fully staffed and equipped mobile clinics made monthly visits to major sites where mine workers meet SWs | Not stated |
| 38 | South Africa, Durban-Joburg highway, truck stops[[12](#_ENREF_12), [111-119](#_ENREF_111)] | 1996 | Dedicated clinic | FSWs at 5 truck stops on road between Johannesburg and Durban | Not stated |
| 39 | South Africa, Free State, Virgina town, Harmony Mine[[120](#_ENREF_120), [121](#_ENREF_121)] | 1996 | Dedicated mobile clinic | Mobile clinic providing special services targeting FSWs | Monthly visits scheduled |
| 40 | South Africa, Mpumalanga[[122](#_ENREF_122)] | 1997 | Outreach projects, with peer educators. | 35 projects within the province. These seek to promote HIV awareness and behavioural change through a peer process of condom promotion, STD prevention and HIV risk awareness. | Not stated |
| 41 | South Africa, Pretoria[[123](#_ENREF_123), [124](#_ENREF_124)] | 2000 | Field office specifically for study participants. | Black South African sex workers working in daily rate hotels, apartments, and informal settlements; women working the streets of Pretoria were also recruited. Cocaine use or a positive urine cocaine test were other specific eligibility criteria. | Not stated |
| 42 | Southern Africa, Durban-Lusaka highway, Northern Province highways [[125](#_ENREF_125)] | Not stated | District hospital and two public clinics. Outreach for FSWs and high-risk groups | Target high-risk populations at borders, highways | Not stated |
| 43 | Mozambique, all provinces[[126](#_ENREF_126), [127](#_ENREF_127)] | 1994 | Stand-alone service delivery model. | Stand-alone service delivery model. Condom social marketing targeting truckers, military, FSWs | Not stated |
| 44 | Tanzania, highway [[128](#_ENREF_128)] | 1989 | Peer educators services for FSWs and truck drivers at truck stops | Seven truck stops on highway from Dar es Salaam to Zambia – these truck stops already in an existing HIV prevention programme, 330 high-risk women. 3 trucking companies in Dar es Salaam | follow up from bi-weekly to 3 monthly |
| 45 | The Gambia. Banjul, Serrakunda, small towns on TransGambia Highway (Farafenni and Soma), Basse (cross-border site)[[129-133](#_ENREF_129)] | 1989 | Dedicated clinics and outreach by field workers who contacted FSWs at their workplace or home. | Three Medical Research Council clinics targeting FSWs | Not stated |
| 46 | Togo, Lome and other urban centres [[134](#_ENREF_134)] | 1990 | Research clinic for FSWs and clients | A clinic in the capital city of Lome, and other towns in Togo | Daytime |
| 47 | Uganda, Kampala, Kibuye [[135](#_ENREF_135)] | 2008 | Stand-alone clinic offering free general and reproductive health care. | On-site laboratory located in a slum | Not stated |
| 48 | West Africa, highways [[136](#_ENREF_136)] | 2001 | Community outreach for HIV/AIDS care and support. Integrated services – all major public and private health facilities along the transport corridor. | Abidjan-Lagos transport corridor across five countries, with major truck-stops and borders | Not stated |
| 49 | West Africa: Benin, Burkina Faso, Ghana, Mali, Niger, Senegal, Togo[[137](#_ENREF_137)] | Not stated | National programs were designed as an integrated approach, combining adapted services. Community outreach in collaboration not only with public services, but also with private health structures. | Sex workers, their clients and the larger population of women and men living in environments where sociosexual networks may contribute to the propagation of infection. | Not stated |
| 50 | Zambia, Livingstone, border towns and corridor communities [[138](#_ENREF_138)] | 2000 | Clinics and mobile units for FSWs and truck drivers. Mobile units provide STI and HIV | Facility centrally located in a nondescript house | Not stated |
| 51 | Zimbabwe, Bulawayo[[69](#_ENREF_69), [139](#_ENREF_139)] | 1989 | Peer services in homes, bars, and streets | Program for sexually vulnerable groups in Bulawayo, Zimbabwe's. FSWs and vulnerable groups of men, including clients, bar patrons, military, truckers, and migrant workers. | Not stated |
| 52 | Zimbabwe, Harare[[140](#_ENREF_140), [141](#_ENREF_141)] | Not stated | Study clinic set up for only FSWs | Targeting FSWs | Not stated |
| 53 | Zimbabwe, West Mashonaland, commercial farms and mines[[69](#_ENREF_69), [141-144](#_ENREF_141)] | 2000 | A research clinic was set up for sex workers only | Research clinic set up at each rural workplace site (5 farms and 2 mines) | Nurse at clinic for several hours from Monday to Friday |
| 54 | Zimbabwe, Shurugwi, Midlands mining town[[145](#_ENREF_145)] | 1988 | Services at 2 hospitals, referral sites of mining clinics | FSWs in Shurugwi (rural town with mining compounds). | Not stated |

**Supplementary Table 2: Package of clinical services provided in African sex worker projects**

|  | **Country, City** | **Family planning** | **Male and female condoms, safe sex** | **HIV T&C, CD4 testing, care and ART** | **RTI services (screening, syndromic management and PPT)** | **Cervical cancer** | **GBV services** | **Other services** |
| --- | --- | --- | --- | --- | --- | --- | --- | --- |
| 1 | Benin, Cotonou, Porto Novo, 10 small town clinics across country [[1-13](#_ENREF_1)] | Urine pregnancy tests | Condom promotion and FSW given free condoms and clients given 20 condoms. Correct condom use part of intervention | HIV-1 and HIV-2 T&C | Intensive STI education. Genital exam by doctor or midwife with speculum visualization of cervix (noting presence of ulcers, inguinal adenopathy, warts, vaginal and cervical discharge, and pain on cervix mobilization). Vaginal and cervical samples collected, pus on cervical swab noted. Blood and first urine samples taken. Monthly STI screen and treat. Screening for NG, CT, M genitalium. Syphilis, BV, TV and candida. Cervical swab and urine for NG testing. STI management and screening used algorithms. Free STI management. Syndromic approach as in national guidelines. PPT trial azithromycin 1g 1st month and Ciprofloxacin 500mg 2nd and 3rd month. Male clients had genital exam and given syndromic treatment, for symptoms or if leucocyte esterase dipstick test positive | Not stated | Not stated | IEC, educational campaigns on HIV/AIDS/STIs |
| 2 | Burkina Faso, Bobo-Dioulasso [[9](#_ENREF_9), [14-18](#_ENREF_14)] | Not stated | Peer-led education sessions on condom use (including negotiation skills) at each 3-monthly follow-up visit. Condom promotion and distribution at each visit. | HIV 1 and 2 T&C, and information and education on HIV testing and information at enrolment and f/u visits. Peer-led education sessions on HIV prevention and HIV disease progression. Since 2004, ART provided, following WHO guidelines. Adherence support. If women miss visits, telephone calls and home visits made by peer-educators and social workers. Cotrimoxazole given. Tried to decrease stigma and discrimination by following FSWs and non-FSWs receiving ART together at same clinic | Pelvic exam. Genital and blood samples taken for syphilis, NG, CT, TV BV, candida, HSV-2, and, in the case of ulcer, haemophylius ducreyi. Tests repeated at 3-monthly follow-up visits. Partner tracing offered, though partners difficult to reach. Syndromic management. Peer-led education sessions on STI prevention | Not stated | Not stated | Consultation with a psychologist for either HIV pre-test counselling or support. All medical care, including investigations and treatment, provided for free. |
| 3 | Cameroon, Yaounde and Douala [[19-22](#_ENREF_19)] | Not stated | Provision of free condoms, and advice on condom use | HIV T&C monthly f/u. HIV-infected referred to 'other services' | STIs history, pelvic exam and speculum or colposcope. NG, TV and vaginal wet mount tests. Women positive for non-viral STIs contacted and treated. NG positive also treated for CT. Blood taken for syphilis tests 3 monthly | Not stated | Not stated | Free medical care for study participants. |
| 4 | Cote d' Ivoire, Abidjan, and 11 other towns [[23-33](#_ENREF_23)] | Urine pregnancy tests | Condom promotion and provision, including female condom and lubricating gel. Education activities by peer workers, including picture album tool, video films, drawings for education, condom demonstration and provision, group education in community | HIV T&C, care services | Free STI diagnosis and treatment (follow local guidelines, STI treatment algorithms), fully equipped, staffed by 5 physicians. Pelvic exam with speculum and/or cervical sampling. In trial, STI screening with monthly speculum exam, direct micro wet prep. Syphilis serology, candida diagnosis, endocervical swabs | Not stated | Not stated | Health education messaging. PHC services |
| 5 | Democratic Republic of Congo, Kinshasa, Matonge [[34](#_ENREF_34)] | Not stated | Condom promotion group sessions every 3 months. Monthly intensive individual health education and free condoms | 3 monthly HIV 1 &2 TC, risk reduction counselling | Monthly STI screening & free treatment. Clinical exam, including pelvic exam and cervical swab collection by physician. NG, CT, TV, candida, syphilis, HSV-2 testing, monthly at times. Syndromic management, using WHO algorithms. Patients return after 1 week for additional treatment if indicated. Studies demonstrated weaknesses in STI screening and syndromic management of STIs, which missed many cases. | Not stated | Not stated | Health education |
| 6 | East and Central African highways [[35](#_ENREF_35)] | Peer-based family planning (not specifically for FSWs) | Not stated | Not stated | Not stated | Not stated | Not stated | Peer-based health education, and referrals (not specifically for FSWs) |
| 7 | Ghana, Accra [[36](#_ENREF_36)] | Not stated | Not stated | HIV T&C in temporary base | STI education | Not stated | Not stated | Peer educators give health promotion to FSWs and their non-paying partners (pimps) in their own homes |
| 8 | Ghana, Accra, Kumasi, Techiman [[5](#_ENREF_5), [37-42](#_ENREF_37)] | Not stated | Condoms sold during site visits or at clinics. Also condom promotion and distribution to peer educators for subsequent distribution to their peers. In a trial, all FSWs given free condoms and clients 20 condoms | HIV 1 and 2 testing | Syndromic management on cost recovery basis. 4 clinic visits/year for STI screening, drugs free. Gynaecologic exam by doctor or midwife, with speculum. Cervical swab tested for pus, NG and CT, Mycobacterium genitalium and TV. PPT trial ciprofloxacin and doxycycline, azithromycin | Not stated | Not stated | Information and education on HIV and STIs. Group meetings in SW communities, bars and clinic for continued education sessions conducted by the trainer, physician and outreach staff. Treatment of other health problems on cost recovery basis |
| 9 | Guinea, Conakry [[43](#_ENREF_43)] | Not stated | Condoms sold during site visits or at clinics. Also condom promotion and distribution to peer educators for subsequent distribution to their peers. In a trial, all FSWs given free condoms and clients 20 condoms | HIV 1 and 2 testing | Syndromic management on cost recovery basis. 4 clinic visits/year for STI screening, drugs free. Pelvic exam by doctor or midwife, with speculum. Cervical swab tested for pus, NG and CT, Mycobacterium genitalium and TV. PPT trial ciprofloxacin and doxycycline, azithromycin | Not stated | Not stated | Information and education on HIV and STIs. Group meetings in SW communities, bars and clinic for continued education sessions conducted by the trainer, physician and outreach staff. Treatment of other health problems on cost recovery basis |
| 10 | Kenya, Kisumu [[44](#_ENREF_44)] | Not stated | Not stated | HIV T&C | Syphilis, HSV-2. Endocervical swabs for CT and NG. Vaginal swab for TV, BV | Not stated | Not stated | Not stated |
| 11 | Kenya, Mombasa, Kisauni [[45-49](#_ENREF_45)] | Not stated | Drop-in centre distributed condoms and promotion materials. Peers provided condom promotion in community. | HIV T&C at drop-in centres. Peers accompanied by mobile HIV T&C services | Provided information and referrals for STI treatment | Not stated | Not stated | Peers provided HIV education and other risk-reduction activities. |
| 12 | Kenya, Mombasa, Ganjoni [[50-59](#_ENREF_50)] | Not stated | Free male condoms | HIV T&C each monthly visit. CD4 testing for those HIV infected | Pelvic exam with speculum and collection of STI and RTI screening specimens (NG, CT, TV, BV, candida, H. ducreyi). Syphilis, HHV-8. STI treatment according to national guidelines. Colposcopy | Not stated | Not stated | Risk-reduction counselling. Monthly general physical exam, oro-pharyngeal, chest, abdomen. |
| 13 | Kenya, Nairobi, Mukuru [[60](#_ENREF_60)] | Not stated | Not stated | HIV T&C. HIV-infected referred to ART site | Not stated | Not stated | Not stated | Community education services |
| 14 | Kenya, Nairobi, Majengo Observational Cohort Study (MOCS) [[61](#_ENREF_61)] | Free contraception | Free condoms | Not stated | Free STI care and treatment | Not stated | Not stated | Educate SWs about HIV and other STIs and encourage visits to Majengo clinic and follow-up. Level III bio-containment unit along with expansion of existing infrastructure and capacity related to general bacteriology, virology, serology, and PCR. Health education and 1-one-1 counselling |
| 15 | Kenya, Coast Province, Kilifi District, Mtwapa [[62](#_ENREF_62)] | Not stated | Condoms given and lubricants | Oral pre-exposure prophylaxis for HIV prevention with adherence support | Not stated | Not stated | Not stated | Physical exam |
| 16 | Kenya, Nairobi, Kibera [[63-68](#_ENREF_63)] | Not stated | Free male condoms at clinic, risk-reduction counselling | HIV T&C 3 monthly in HIV prevention trial. On request thereafter. When ART rolled out in 2004, all known positives contacted and informed of services | PPT trial azithromycin, DOT. Physical exam 6 monthly. STI screen for syphilis, NG, CT, TV, BV, H. ducreyi, HSV-2. Syndromic management (Kenyan guidelines). Monthly pelvic exam. | Not stated | Not stated | 2 standardized intensive risk-reduction counselling sessions of 1 hour, with subsequent peer and clinic-based counselling. |
| 17 | Kenya, Nairobi, Pumwan, Majengo[[69-73](#_ENREF_69)] | Not stated | Male condom distribution at clinics and by community workers. Condom promotion at meetings and individual counselling includes training on supply sources, storage, proper use and disposal, rejection of potential clients who refuse to use condoms | HIV T&C every 6 months. CD4 tests | Scheduled clinic visits for exam and STI treatment (initially 2 weekly, later longer intervals) or when needed | Not stated | Not stated | General clinical services available. Microfinance provided credit and business skills training to FSWs. Loan fund for small businesses. Mid-stream urine. Patients with bateriuria and UTI treated with antibiotics |
| 18 | Kenya, trans-Africa highway[[74](#_ENREF_74)] | Not stated | Not stated | HIV prevention campaign materials | Not stated | Not stated | Not stated | Not stated |
| 19 | Madagascar, Antananarivo, Tamatave, Antsiranana, Mahajanga, Toamasina[[75-86](#_ENREF_75)] | Methods include diaphragm. Information given about emergency contraception, sold for $1 from clinic. (low use by women). Plans to offer free hormonal contraception. Pregnancy testing done | Safe sex counselling and instructed to use condoms at all acts. Given some male condoms, advised to purchase additional condoms in community from peer educators or social marketing agents and outlets. In some years, given all condom supplies. Bimonthly clinic counselling by physician/nurse for 6 months, 15min 2-way info exchange about dual protection; demonstration of male and female condoms; practice with anatomical models; skills for negotiating condom use; and promotion of ‘‘no condom=no sex’’ policy. Bimonthly condom promotion and risk reduction counselling by peer educators in clinic and community. Peer educators trained in social marketing, sold condoms at lowest price available in that city. Female condoms only advised for when male condom couldn't be used. Female condoms 20% of final condom use | HIV T&C. Individual education on HIV/STI treatment & prevention | Innovative STI services. User-centred STI care (including peer educator referral). Screening for syphilis, TV, candida, BV, NG and CT. Bimanual & speculum exam with swab collection. Treatment following STI guidelines. PPT with azithromycin and ciprofloxacin, also PPT for TV and BV. Counselled about STI treatment and prevention. If LAP, metronidazole given. At 3-monthly f/u visits, lab & clinical evaluations repeated. STI care free. STI case management includes referral for partner treatment. Improved basic STI services for SWs in partnership with SWs, by using participatory action research. STI services for 1st clinic attendance different from subsequent visits, which was risk-score based | Not stated | Not stated | National STI guidelines developed in these sites. Clinic and community-based peer-risk reduction counselling. UTI screening with urinanalysis. Bi-monthly clinic counselling by physician/nurse for 6 months, 15min 2-way info exchange about individual risk assessment; basic knowledge about STI/HIV |
| 20 | Madagascar, Diego-Suarez[[87](#_ENREF_87)] | Not stated | Clinic- and community-based education in order to promote consistent condom use | Not stated | STI clinic, recommended monthly visits for STI care and prevention, changed to every 3 months (treatment 3 monthly is risk based). Cervical infection based on incorporated laboratory and individual risk information. Clinic- and community-based education in order to promote consistent condom use and STI care seeking among SWs. Speculum exam. Lab/risk-based treatment (only in Diego-Suarez, not national), with treatment only if fulfil criteria. PPT at first visit, risk based treatment at 3-monthly f/u visits | Not stated | Not stated | Programme to register FSWs and ensure they attend STI clinic else not given access to the port area. |
| 21 | Malawi, 3 towns in Thylo District (Thyolo, Luchenza and Bvumbe)[[88](#_ENREF_88), [89](#_ENREF_89)] | Not stated | Female condoms provided, with counselling and practical demonstration. IEC sessions on condom use provided by district health team | Not stated | District health team do weekly STI screening, management using national STI guidelines. Genital examination, including speculum. Patient card contained details of STI management. FSWs issued with medical clearance certificate after screening/treatment | Not stated | Not stated | Not stated |
| 22 | Malawi, Dowa and Lilongwe Districts [[90](#_ENREF_90)] | Not stated | Peer education interventions to teach safe sex negotiations skills to FSWs, condom promotion and distribution | Peer educators trained to give info about HIV (had difficulties doing so) | Not stated | Not stated | Not stated | Health talks given by health staff to FSWs |
| 23 | Malawi[[91](#_ENREF_91)] | FP services offered - not specified which | Free condom distribution at 16 sites with easy access by FSWs: toilets, residential areas, clubs. DJs distributed safe sex messages | HIV T&C, no mention of referral | STI services were offered through Youth Life Centres at places of entertainment | Not stated | Not stated | IEC materials provided, messages also designed to encourage young SWs to withdraw from SW and opt for other ways of earning a living. Information on HIV shared via presentations, discussions and testimonies. Sex worker Safer Sex Kit distributed. 6 sex workers set up tailoring business |
| 24 | Mali, Bamako[[43](#_ENREF_43)] | Not stated | Not stated | Not stated | Initial visit screening for NG/CT/TV using cervical swabs, pelvic and speculum exam, PPT (all women given 2g metronidazole & cotrimazole vaginal cream for 3 days). FSWs return 7 days later | Not stated | Not stated | Not stated |
| 25 | Mozambique, Tete, Moatize[[92](#_ENREF_92)] | Contraceptive services | Condom distribution by peer educators trained in behaviour change communication | Education and communication on HIV; HIV T&C | STI care and information; syphilis testing | Not stated | Not stated | None |
| 26 | Namibia, Oshakati[[93](#_ENREF_93)] | Not stated | Condom Social Marketing Scheme. Including condom demonstrations and distribution | Not stated | Not stated | Not stated | Not stated | Behavioural change communication sessions. |
| 27 | Namibia, Katima Mulilo, Oshikango, Windhoek, Keetmanshoop, Walvis Bay and Swakopmund [[94](#_ENREF_94)] | Not stated | Not stated | Not stated | Not stated | Not stated | Not stated | Comprehensive HIV prevention services and linkages to care and treatment. Referral networks established in towns where programme is implemented. Package of HIV prevention interventions for each target population and training and sensitization for stigma reduction and improvement of care and support |
| 28 | Nigeria, Asawara state[[95](#_ENREF_95)] | Not stated | Not stated | HIV T&C. No mention of referral to ART | Syphilis testing | Not stated | Not stated | Physical examination |
| 29 | Nigeria, Cross River State, Calabar and Ikom. Also Port Harcourt, Rivers State | Not stated | Condom promotion and distribution by peer educators. Condoms free initially, then sold at subsidized price while emphasising that condoms save money (such as for prophylactic antibiotics) | Monthly ''chairladies meetings'' with FSWs discussion HIV prevention. HIV T&C | STI clinic for clients and FSWs. FSW referrals by "chairladies", free clinic registration cards, posters with clinic hours/dates at hotels. Client referrals by FSWs and managers. Aimed at STD diagnosis and treatment. Laboratory and clinical diagnosis of STDs offered. PPT for STIs. | Not stated | Not stated | Assisted with fumigation of hotel premises, with child welfare issues. Health education and counselling services on sexual behaviour |
| 30 | Nigeria, Jos[[97](#_ENREF_97)] | Not stated | Not stated | HIV T&C. HIV-infected FSWs referred to facility in Jos for ART, care and support | Pelvic exam by gynaecologist. High vaginal and endocervical swabs for TV, candida, BV, NG. Syphilis testing. Free treatment for treatable STIs | Not stated | Not stated | Not stated |
| 31 | Rwanda, Kigali[[98-101](#_ENREF_98)] | Given pregnancy prevention counselling, and 3-monthly pregnancy testing | Not stated | HIV T&C 3 monthly. If positive, CD4 test done and referred to local health centre for care, ART, PMTCT if pregnant, and psychosocial services (in some instances, FSWs concerned about potential unintentional HIV disclosure to community members, were referred to more distant sites) | Testing for HSV-2, Syphilis, BV, candidiasis, TV, CT, NG. Pelvic exam with collection of vaginal and cervical specimens, 6 monthly. Treatment given for curable STIs using Rwanda’s National Guidelines for syndromic management of STIs/RTIs and WHO guidelines. Partner notification done | Not stated | Not stated | Referral for other services, if required |
| 32 | Senegal, Dakar [[146](#_ENREF_146)] | Not stated | Free condoms. Demonstration classes | HIV T&C yearly | Physical and gynaecological exam, 2-monthly lab tests for NG, CT, TV, syphilis, other vaginal bacterial infections). Medication provided free. Programme includes full medical check, swabs every 2 months, medical exam alternate months. Required to register as sex workers and have regular STI screening | Not stated | Not stated | Education on safe sex, STIs and contraceptives at consultation. 1969 Law required self-identified FSWs to enrol for monthly medical follow up and examination. Sex educational and HIV awareness classes ≥3/week at clinic, run by SWs or presenter from Association for Women at risk from AIDS, often a peer-educator |
| 33 | Senegal, Dakar, Kaolack, Ziguinchor and Saint-Louis [[147](#_ENREF_147), [148](#_ENREF_148)] | Not stated | Free condom promotion and provision. Counselling and promotion of safe behaviour and condom usage among FSW (social campaigns in public/private areas, many provided by NGO's) | Awareness campaigns for responsible and safer sex. HIV 1 and 2 T&C | Widespread screening and treatment of STIs (package in place since 1969) for FSW. Mostly integrated within existing PHC facilities. Screening for syphilis, NG, CT and TV and, HD and candida | Cervical Pap smear and treatment of cervical lesions | Not stated | Health education provided |
| 34 | Sierra Leone, Port Loko[[103](#_ENREF_103)] | Not stated | Free condom distribution during community outreach. Also organised distribution by bar owners and peer educators | Not stated | Not stated | Not stated | Not stated | Widespread IEC activities |
| 35 | South Africa, Johannesburg, Hillbrow[[12](#_ENREF_12), [104-108](#_ENREF_104)] | Family planning advice and urine pregnancy testing | Male and female condom distribution, together with health education about STIs and safe-sex negotiation skills, all provided by CHWs during outreach. Women instructed on reuse of female condom, given lubrication for this. Counselled on safer sex. Safe-sex negotiation skills | HIV T&C in outreach | Pelvic exam and STI screening in permanent clinic and mobile clinic outreach: candida, CT, NG, TV and Syphilis testing. A study assessed syndromic management and PPT (azithromycin) for FSWs in clinic. Women attended monthly clinic visits. Mobile STI clinic to hotels about 3 days per month, operated by nurse and CHW | PAP smears | Not stated | Health education. Referral network for social welfare needs. |
| 36 | South Africa, Cape Town [[109](#_ENREF_109)] | Not stated | Condom distribution by FSW fieldworkers. Taught by SWEAT to eroticize condom use (but discontinued this approach). Condoms free at clinics or distributed by team | Interviewers provided AIDS education and distributed HIV educational material. Training workshops skill FSWs in passing on HIV education and materials to clients, and discussing safe sex | Interviewers distributed STI educational material | Not stated | Not stated | NGO built a working relationship with clinics, provided training to staff, who then accompanied fieldworkers on outreach visits. To encourage FSWs to be more open about their work at clinic. NGO and community lobbied for more public cleaning and toilet facilities. Initiated intervention with gatekeepers (pimps or gangs), so they support and accompany FSWs to drop-in centre. Gatekeepers/pimps included in health education programs. Pimps given educational material and condoms, protect outreach health educators on late night shifts |
| 37 | South Africa, Carletonville[[110](#_ENREF_110)] Mothusimpilo | Not stated | Peer education and distribution of condoms | Not stated | Trained health workers in syndromic management of STI, provided these services. FSWs given directly-observed PPT (azithromycin) | Not stated | Not stated | community meetings |
| 38 | South Africa, Durban-Joburg highway, truck stops[[12](#_ENREF_12), [111-119](#_ENREF_111)] | Not stated | Free condoms, education, intensive counselling. Promotion of condom use at all sex acts | HIV T&C (monthly at times) | Monthly screening and treatment for TV, CT, NG, candidiasis. Also syphilis, H. ducreyi, BV, HSV and HPV testing. Clinical and pelvic exam, including speculum. STIs treated according to South African syndromic management guide | Pap smears, colposcopy | Not stated | Efforts to improve literacy. Educational programs |
| 39 | South Africa, Free State, Virgina town, Harmony Mine[[120](#_ENREF_120), [121](#_ENREF_121)] | Not stated | Condom promotion, distribution & IEC | Not stated | Genital exam, including speculum. Monthly PPT (azithromycin). Syphilis, NG, CT testing. Syndromic management | Not stated | Not stated | Prevention education |
| 40 | South Africa, Mpumalanga[[122](#_ENREF_122)] | Not stated | Since 1997, peer education based health program of male condom promotion to promote HIV awareness and behavioural change. 120,000 FC distributed by peer educators trained in FC use | Peer educators promote HIV risk awareness | Peer educators promote STD prevention | Not stated | Not stated | Not stated |
| 41 | South Africa, Pretoria[[123](#_ENREF_123), [124](#_ENREF_124)] | Not stated | Demonstration and rehearsal of male and female condom use. Cue cards used to provide info on sexual risks; risk-reduction methods including proper use of male and female condoms; how to discuss safer sex practices. Personalized assessment of each FSW’s sexual risks, goals for negotiating risk-reduction by communicating the importance of condom use | Cue cards used to provide information on HIV, HIV prevention and T&C | Not stated | Not stated | Not stated | Private 1-hour education and skills-building sessions. Cue cards used to provide information on drug risks. Given a risk-reduction and toiletry kit, info on referral resources. Personalized assessment of each woman’s drug and sexual risks. Women learned violence prevention strategies such as staying sober, communication techniques in difficult situations, and exiting a volatile situation. FSWs shown how to seek community resources. Intervention emphasised contextual and cultural barriers to increased condom use and lifestyle, including development of personal concrete plans to support goals of reducing risks and developing more independence. Role-played and rehearsed verbal assertiveness. |
| 42 | Southern Africa, Durban-Lusaka highway, Northern Province highways [[125](#_ENREF_125)] | Not stated | Targeted peer education project provides condoms. Also other commercial and public sector condom outlets, socially marketed condoms | Not stated | Local clinic and district staff trained in syndromic management., use syndromic management flowcharts | Not stated | Not stated | Not stated |
| 43 | Mozambique, all provinces[[126](#_ENREF_126), [127](#_ENREF_127)] | Not stated | Condom social marketing. Behaviour-change activities targeting FSWs, traders, nightclub patrons, truckers, police/military, migrant labour and STI clinic attendees. Male condoms distributed | Not stated | SRH services provided | Not stated | Not stated | IEC campaign through educational materials, street theatre, TV, radio to promote JeitO branded condoms. Behaviour change such as peer education debates and theatre |
| 44 | Tanzania, highway [[149](#_ENREF_149)] | Not stated | Peer educators provide condom promotion and education; teach correct use, negotiation skills and condom social marketing skills. In 1990, Ministry of Health distributed condoms only at hospitals, and often unavailable through commercial outlets. Project extended condom distribution into hotel bedrooms, bars and company bathrooms. Project later complemented by other distribution systems, including social marketing systems. Well-packed and marketed condoms were believed to be better quality than free distributed condoms and were soon preferred by customers. Verbal messages were reinforced. Most messages targeted condom use with 'casual' unknown partners | HIV T&C. AIDS prevention program: truckers, assistants, FSW peer education (for behaviour change, and HIV transmission and prevention counselling) | Since 1989, targeted truck drivers, assistants, FSW services included promotion of appropriate STD care-seeking behaviour. Since 1993 targeted mainly female partners of truck drivers and added confidential STI services. Several STI treatment delivery modes: Primary Health Care worker outreach clinic twice/ week (outside normal clinic hours at clinic or at site chosen by FSWs); Primary Health Clinic with STI drugs during normal working hours and with other patients; Doctor-led outreach clinic 3 monthly (STI diagnosis and treatment out of facility). Local clinicians trained for 1 week in STI management, WHO algorithms & risk assessment. Syndromic treatment used. Asymptomatic STIs treated on risk. Peer educators trained for 1 week to assess STI risk and recognize signs & symptoms of STD, and if present, to issue referral card. Blood for syphilis and CT antigen. Vaginal and endocervical swabs for wet mount microscopy, TV diagnosis, and NG culture. Treatment if clinical/lab evidence. General and pelvic exam. Peer educators encouraged health-seeking behaviour for STIs. | Not stated | Not stated | Not stated |
| 45 | The Gambia. Banjul, Serrakunda, small towns on TransGambia Highway (Farafenni and Soma), Basse (cross-border site)[[129-133](#_ENREF_129)] | Not stated | Male condoms provided. Counselling on condom use | HIV 1 and 2 T&C | Screening for CT, NG, H. drucreyi. Physical exam, speculum and swabs taken. STIs treated according to standard protocols | PAP smear | Not stated | Health education about HIV, STIs. Free health care at MRC clinics |
| 46 | Togo, Lome and other urban centres [[134](#_ENREF_134)] | Not stated | Peer educator distribution. Condoms supplied to clients and FSW | Clients and FSW HIV T&C. Referrals for those HIV positive | NG and CT. Free STI treatment for clients and FSW | Not stated | Not stated | Preventive and curative care to SWs and clients |
| 47 | Uganda, Kampala, Kibuye (densely populated slum) [[135](#_ENREF_135)] | FP offered | Free condoms provided at clinic | HIV T&C. CD4 and complete blood, liver and renal function testing for HIV+ve women. Pre-counselling for ART at study clinic, accompanied to HIV-care centre for ART initiation. Cotrimoxazole prophylaxis given | 3-monthly HSV-2 and syphilis, gynae exam, including for STI syndromes. NG, CT, TV, BV, Candida testing. If genital ulcers present, swabs taken and tested. Syndromic management as per Uganda guidelines and followed up once STI aetiology determined | Not stated | Not stated | Health education and counselling at clinic. Free access to the general care clinic. Participants children <5Y also access free care |
| 48 | West Africa, highways [[136](#_ENREF_136)] | Not stated | Social marketing of condoms (male and female) in eight border communities and along transport corridor, 625 sales points manned often by FSWs (trained by project). 20 automatic condom dispensers. Project produced own condom #The MIGRANT# and #FEMIGRANT# distributed in kiosks put in place along corridor. Condoms distributed to commercial vehicle drivers, FSWs and by social marketing. Promotion of condom use through - T-shirts, calendars, caps, bags, billboards, radio commercials, etc.(localizing adverts to decrease cost) | Integrated IEC and BCC program for HIV prevention. Messages broadcast by 32 radio networks. FSWs, local population, vehicle drivers taught ≥2 ways to prevent HIV. Special information days initiated. Annual rally at border crossing to mark World AIDS day, involving residents and vehicle drivers. Information and sensitization campaigns on HIV (called Love Life Caravans) organized, approx 3mil people reached. HIV treatment, care, support strengthening public and private health care facilities, VCT and treatment of HIV opportunistic infections. Financed treatment of ARVs for about 500 people, ARV referral system in place. ARVs principally to vehicle drivers testing positive at border towns. Since '06 and '07, ARVs free, except Ghana. Doubled number of VCT centres, at least one VCT centre on either side of border, with IEC campaigns. Inter-country HIV coordination amongst governments and project stakeholders. Health care providers trained in VCT, psychological care and support, ART, PMTCT, laboratory. Intense IEC activities to increase HIV knowledge by peer educators. | Health facilities offer STI diagnosis and treatment. IEC/BCC materials for STI awareness. Facilities given STI kits, reagents, and audio/visual material. Staff training in STI syndromic management. Strengthening public and private facilities along the corridor to provide STI treatment | Not stated | Not stated | 36 public, private and community-based health facilities were upgraded, equipped and provided with an adequate supply of drugs and other pharmaceuticals. Staff in these facilities was trained. The project renovated 16 facilities close to the 8 border posts to provide VCT services. 2) implementation of transport sector policies aimed at expediting border crossing and removing informal barriers to the movement of people and goods,to reduce the importance of sex markets at border towns. Helped establish the Association of Professional Truckers and Drivers, played an important role in disseminating messages on HIV transport facilitation3) Project was fully consistent with the MAP2 eligibility criteria for sub-regional projects, fully aligned with RIAS, background analysis of transoprt corridor adequate, lessons learned from international experience in fighting HIV incorporated, objective well defined, institutional framework elaborate, strong commitment from 5 governments, risk analysis adequate. 4) Waste management plan - 9 incinerators and 9 septic tanks installed, staff trained 5) A number of training programs and sessions held- for dissemination of information by media, health care waste management, principles of free movement of goods, and people, observatory practices at borders, financial management. 6) Blood transfusion facilities also received reagents and consumables 7) Localized radio channels - over 30 million sensitized. MF: 36 public, private and community-based health facilities were upgraded, equipped and provided with an adequate supply of drugs and other pharmaceuticals. Staff in these facilities was trained. The project renovated 16 facilities close to the 8 border posts to provide VCT services. FSWs were mobilized to protect themselves as well as to adopt alternate means of income by selling the condoms. |
| 49 | West Africa: Benin, Burkina Faso, Ghana, Mali, Niger, Senegal, Togo[[137](#_ENREF_137)] | Not stated | Not stated | Not stated | Programme focused on syndromic STI control, using WHO standards to detect, treat and prevent STIs | Not stated | Not stated | Community participation to encourage activities promoting health and local responses to HIV. Support organizations that offer alternatives and contribute to creating coalitions that promote health. National programs designed as an integrated approach, combining varying degrees of adapted services and community support targeting SW environments |
| 50 | Zambia, Livingstone, border towns and corridor communities [[138](#_ENREF_138)] | Not stated | Condom distribution, behaviour change and communication strategy to raise awareness and knowledge about risky sexual behaviour | HIV T&C, referrals for ART and care for HIV-positive | Targeted STI testing and treatment services in clinics | Not stated | GBV and legal protection through referrals to local police. Violence-related health needs referred to government hospitals | Behaviour change and communication strategy to change risky sexual behaviour. |
| 51 | Zimbabwe, Bulawayo[[69](#_ENREF_69), [139](#_ENREF_139)] | Not stated | Peer educators distributed condoms in their social networks and at bars, selected workplaces and health facilities | no HIV testing available | Free STI treatment cards for use at local public sector facilities, for themselves, and for other SWs | Not stated | Not stated | Health education |
| 52 | Zimbabwe, Harare[[140](#_ENREF_140), [141](#_ENREF_141)] | Not stated | Free male or female condoms. Shown how to use condoms. Given pretested diagrammatic instruction sheets on use of female condoms and asked to practice insertion in the presence of a research nurse | HIV T&C | Offered STI screening and treatment. Pelvic examinations and cervical, vaginal, and blood specimens, tested for NG, TV, CT and syphilis | Not stated | Not stated | Sexual health advice |
| 53 | Zimbabwe, West Mashonaland, commercial farms and mines[[69](#_ENREF_69), [141-144](#_ENREF_141)] | Not stated |  | HIV T&C | Observed PPT (azithromycin, metronidazole, ciprofloxacin). Testing for HSV-2, syphilis, CT, NG, TV. Free syndromic management according to local guidelines. Partner notification. Acyclovir for HSV-2 | Not stated | Not stated |  |
| 54 | Zimbabwe, Shurugwi, Midlands mining town[[145](#_ENREF_145)] | Not stated | Not stated | Referrals for HIV T&C | Medical exam by medical officer. Syphilis, MC&S, Urinalysis. Card holders examined monthly, card withdrawn if positive for STI until it is clear | Not stated | Not stated | Given health card allowing admission to beer halls where SWs get clients (checked by security guards). |

- indicates no services provided, or not applicable; f/u follow up; T&C testing and counselling; PHC primary health care; EC European Community

**References**

1. Mukenge-Tshibaka L, Alary M, Bernier F, van Dyck E, Lowndes CM, Guedou A, Anagonou S, Joly JR: **Diagnostic performance of the Roche AMPLICOR PCR in detecting Neisseria gonorrhoeae in genitourinary specimens from female sex workers in Cotonou, Benin.** *J Clin Microbiol* 2000, **38:**4076-4079.

2. Mukenge-Tshibaka L, Alary M, Lowndes CM, Van Dyck E, Guedou A, Geraldo N, Anagonou S, Lafia E, Joly JR: **Syndromic versus laboratory-based diagnosis of cervical infections among female sex workers in Benin: implications of nonattendance for return visits.** *Sex Transm Dis* 2002, **29:**324-330.

3. Alary M, Lowndes CM, Mukenge-Tshibaka L, Gnintoungbe CA, Bedard E, Geraldo N, Jossou P, Lafia E, Bernier F, Baganizi E, et al: **Sexually transmitted infections in male clients of female sex workers in Benin: risk factors and reassessment of the leucocyte esterase dipstick for screening of urethral infections.** *Sex Transm Infect* 2003, **79:**388-392.

4. Alary M, Mukenge-Tshibaka L, Bernier F, Geraldo N, Lowndes CM, Meda H, Gnintoungbe CA, Anagonou S, Joly JR: **Decline in the prevalence of HIV and sexually transmitted diseases among female sex workers in Cotonou, Benin, 1993-1999.** *AIDS* 2002, **16:**463-470.

5. Pepin J, Labbe AC, Khonde N, Deslandes S, Alary M, Dzokoto A, Asamoah-Adu C, Meda H, Frost E: **Mycoplasma genitalium: an organism commonly associated with cervicitis among west African sex workers.** *Sex Transm Infect* 2005, **81:**67-72.

6. Ahoyo AB, Alary M, Meda H, Ndour M, Batona G, Bitera R, Adjoni C, Medegan VK, Labbe AC, Adjimon T: **[Female sex workers in Benin, 2002. Behavioural survey and HIV and other STI screening].** *Sante* 2007, **17:**143-151.

7. Lowndes CM, Alary M, Gnintoungbe CA, Bedard E, Mukenge L, Geraldo N, Jossou P, Lafia E, Bernier F, Baganizi E, et al: **Management of sexually transmitted diseases and HIV prevention in men at high risk: targeting clients and non-paying sexual partners of female sex workers in Benin.** *AIDS* 2000, **14:**2523-2534.

8. Lowndes CM, Alary M, Labbe AC, Gnintoungbe C, Belleau M, Mukenge L, Meda H, Ndour M, Anagonou S, Gbaguidi A: **Interventions among male clients of female sex workers in Benin, West Africa: an essential component of targeted HIV preventive interventions.** *Sex Transm Infect* 2007, **83:**577-581.

9. Nagot N, Ouedraogo A, Ouangre A, Cartoux M, Defer MC, Meda N, Van de Perre P: **Is sexually transmitted infection management among sex workers still able to mitigate the spread of HIV infection in West Africa?** *J Acquir Immune Defic Syndr* 2005, **39:**454-458.

10. Germain M, Alary M, Guedeme A, Padonou F, Davo N, Adjovi C, Van Dyck E, Joly JR, Mahony JB: **Evaluation of a screening algorithm for the diagnosis of genital infections with Neisseria gonorrhoeae and Chlamydia trachomatis among female sexworkers in Benin.** *Sex Transm Dis* 1997, **24:**109-115.

11. Lowndes CM, Alary M, Meda H, Gnintoungbe CA, Mukenge-Tshibaka L, Adjovi C, Buve A, Morison L, Laourou M, Kanhonou L, Anagonou S: **Role of core and bridging groups in the transmission dynamics of HIV and STIs in Cotonou, Benin, West Africa.** *Sex Transm Infect* 2002, **78 Suppl 1:**i69-77.

12. Van Damme L, Ramjee G, Alary M, Vuylsteke B, Chandeying V, Rees H, Sirivongrangson P, Mukenge-Tshibaka L, Ettiegne-Traore V, Uaheowitchai C, et al: **Effectiveness of COL-1492, a nonoxynol-9 vaginal gel, on HIV-1 transmission in female sex workers: a randomised controlled trial.** *Lancet* 2002, **360:**971-977.

13. Labbe AC, Pepin J, Khonde N, Dzokoto A, Meda H, Asamoah-Adu C, Mayaud P, Mabey D, Demers E, Alary M: **Periodical antibiotic treatment for the control of gonococcal and chlamydial infections among sex workers in Benin and Ghana: a cluster-randomized placebo-controlled trial.** *Sex Transm Dis* 2012, **39:**253-259.

14. Huet C, Ouedraogo A, Konate I, Traore I, Rouet F, Kabore A, Sanon A, Mayaud P, Van de Perre P, Nagot N: **Long-term virological, immunological and mortality outcomes in a cohort of HIV-infected female sex workers treated with highly active antiretroviral therapy in Africa.** *BMC Public Health* 2011, **11:**700.

15. Nagot N, Ouangre A, Ouedraogo A, Cartoux M, Huygens P, Defer MC, Zekiba T, Meda N, Van de Perre P: **Spectrum of commercial sex activity in Burkina Faso: classification model and risk of exposure to HIV.** *J Acquir Immune Defic Syndr* 2002, **29:**517-521.

16. Nagot N, Ouedraogo A, Defer MC, Vallo R, Mayaud P, Van de Perre P: **Association between bacterial vaginosis and Herpes simplex virus type-2 infection: implications for HIV acquisition studies.** *Sex Transm Infect* 2007, **83:**365-368.

17. Damay A, Didelot-Rousseau MN, Costes V, Konate I, Ouedraogo A, Nagot N, Foulongne V, Van de Perre P, Mayaud P, Segondy M: **Viral load and physical status of human papillomavirus (HPV) 18 in cervical samples from female sex workers infected with HPV 18 in Burkina Faso.** *J Med Virol* 2009, **81:**1786-1791.

18. Konate I, Traore L, Ouedraogo A, Sanon A, Diallo R, Ouedraogo JL, Huet C, Millogo I, Andonaba JB, Mayaud P, et al: **Linking HIV prevention and care for community interventions among high-risk women in Burkina Faso--the ARNS 1222 "Yerelon" cohort.** *J Acquir Immune Defic Syndr* 2011, **57 Suppl 1:**S50-54.

19. Weir SS, Feldblum PJ, Zekeng L, Roddy RE: **The use of nonoxynol-9 for protection against cervical gonorrhea.** *Am J Public Health* 1994, **84:**910-914.

20. Weir SS, Roddy RE, Zekeng L, Feldblum PJ: **Nonoxynol-9 use, genital ulcers, and HIV infection in a cohort of sex workers.** *Genitourinary medicine* 1995, **71:**78-81.

21. Visness CM, Ulin P, Pfannenschmidt S, Zekeng L: **Views of Cameroonian sex workers on a woman-controlled method of contraception and disease protection.** *Int J STD AIDS* 1998, **9:**695-699.

22. Zekeng L, Feldblum PJ, Oliver RM, Kaptue L: **Barrier contraceptive use and HIV infection among high-risk women in Cameroon.** *AIDS* 1993, **7:**725-731.

23. Ghys PD, Diallo MO, Ettiegne-Traore V, Satten GA, Anoma CK, Maurice C, Kadjo JC, Coulibaly IM, Wiktor SZ, Greenberg AE, Laga M: **Effect of interventions to control sexually transmitted disease on the incidence of HIV infection in female sex workers.** *AIDS* 2001, **15:**1421-1431.

24. Ghys PD, Jenkins C, Pisani E: **HIV surveillance among female sex workers.** *AIDS* 2001, **15 Suppl 3:**S33-40.

25. Diallo MO, Ghys PD, Vuylsteke B, Ettiegne-Traore V, Gnaore E, Soroh D, Kadjo JC, Van Dyck E, De Cock KM, Greenberg AE, Laga M: **Evaluation of simple diagnostic algorithms for Neisseria gonorrhoeae and Chlamydia trachomatis cervical infections in female sex workers in Abidjan, Cote d'Ivoire.** *Sex Transm Infect* 1998, **74 Suppl 1:**S106-111.

26. Vuylsteke B, Ghys PD, Mah-bi G, Konan Y, Traore M, Wiktor SZ, Laga M: **Where do sex workers go for health care? A community based study in Abidjan, Cote d'Ivoire.** *Sex Transm Infect* 2001, **77:**351-352.

27. Vuylsteke B, Semde G, Sika L, Crucitti T, Ettiegne Traore V, Buve A, Laga M: **HIV and STI prevalence among female sex workers in Cote d'Ivoire: why targeted prevention programs should be continued and strengthened.** *PLoS One* 2012, **7:**e32627.

28. Vuylsteke B, Traore M, Mah-Bi G, Konan Y, Ghys P, Diarra J, Laga M: **Quality of sexually transmitted infections services for female sex workers in Abidjan, Cote d'Ivoire.** *Trop Med Int Health* 2004, **9:**638-643.

29. Vuylsteke BL, Ettiegne-Traore V, Anoma CK, Bandama C, Ghys PD, Maurice CE, Van Dyck E, Wiktor SZ, Laga M: **Assessment of the validity of and adherence to sexually transmitted infection algorithms at a female sex worker clinic in Abidjan, Cote d'Ivoire.** *Sex Transm Dis* 2003, **30:**284-291.

30. Vuylsteke BL, Ghys PD, Traore M, Konan Y, Mah-Bi G, Maurice C, Soroh D, Diarra JN, Roels TH, Laga M: **HIV prevalence and risk behavior among clients of female sex workers in Abidjan, Cote d'Ivoire.** *AIDS* 2003, **17:**1691-1694.

31. Ettiegne-Traore V, Ghys PD, Maurice C, Hoyi-Adonsou YM, Soroh D, Adom ML, Teurquetil MJ, Diallo MO, Laga M, Greenberg AE: **Evaluation of an HIV saliva test for the detection of HIV-1 and HIV-2 antibodies in high-risk populations in Abidjan, Cote d'Ivoire.** *Int J STD AIDS* 1998, **9:**173-174.

32. Ghys PD, Fransen K, Diallo MO, Ettiegne-Traore V, Coulibaly IM, Yeboue KM, Kalish ML, Maurice C, Whitaker JP, Greenberg AE, Laga M: **The associations between cervicovaginal HIV shedding, sexually transmitted diseases and immunosuppression in female sex workers in Abidjan, Cote d'Ivoire.** *AIDS* 1997, **11:**F85-93.

33. Mastro TD: **Increase in condom use and decline in HIV and sexually transmitted diseases among female sex workers in Abidjan, Cote d'Ivoire, 1991-1998, by Ghys et al.** *AIDS* 2003, **17 Suppl 4:**S121-122.

34. Mann JM, Nzilambi N, Piot P, Bosenge N, Kalala M, Francis H, Colebunders RC, Azila PK, Curran JW, Quinn TC: **HIV infection and associated risk factors in female prostitutes in Kinshasa, Zaire.** *Aids* 1988, **2:**249-254.

35. Morris CN, Morris SR, Ferguson AG: **Sexual behavior of female sex workers and access to condoms in Kenya and Uganda on the Trans-Africa highway.** *AIDS Behav* 2009, **13:**860-865.

36. Papworth V: **Screening hits the streets.** *Nurs Stand* 2009, **24:**24-25.

37. Cote AM, Sobela F, Dzokoto A, Nzambi K, Asamoah-Adu C, Labbe AC, Masse B, Mensah J, Frost E, Pepin J: **Transactional sex is the driving force in the dynamics of HIV in Accra, Ghana.** *AIDS* 2004, **18:**917-925.

38. Adu-Oppong A, Grimes RM, Ross MW, Risser J, Kessie G: **Social and behavioral determinants of consistent condom use among female commercial sex workers in Ghana.** *AIDS Educ Prev* 2007, **19:**160-172.

39. Asamoah-Adu A, Weir S, Pappoe M, Kanlisi N, Neequaye A, Lamptey P: **Evaluation of a targeted AIDS prevention intervention to increase condom use among prostitutes in Ghana.** *Aids* 1994, **8:**239-246.

40. Asamoah-Adu C, Khonde N, Avorkliah M, Bekoe V, Alary M, Mondor M, Frost E, Deceuninck G, Asamoah-Adu A, Pepin J: **HIV infection among sex workers in Accra: need to target new recruits entering the trade.** *J Acquir Immune Defic Syndr* 2001, **28:**358-366.

41. Deceuninck G, Asamoah-Adu C, Khonde N, Pepin J, Frost EH, Deslandes S, Asamoah-Adu A, Bekoe V, Alary M: **Improvement of clinical algorithms for the diagnosis of Neisseria gonorrhoeae and Chlamydia trachomatis by the use of Gram-stained smears among female sex workers in Accra, Ghana.** *Sex Transm Dis* 2000, **27:**401-410.

42. Akumatey B, MacQueen KM, Guest G: **Condom use and HIV prevention among female sex workers in Tema, Ghana. Abstract no. C11518.** 2004.

43. Pepin J, Deslandes S, Khonde N, Kintin DF, Diakite S, Sylla M, Meda H, Sobela F, Asamoah-Adu C, Agyarko-Poku T, Frost E: **Low prevalence of cervical infections in women with vaginal discharge in west Africa: implications for syndromic management.** *Sex Transm Infect* 2004, **80:**230-235.

44. Vuylsteke B, Vandenhoudt H, Langat L, Semde G, Menten J, Odongo F, Anapapa A, Sika L, Buve A, Laga M: **Capture-recapture for estimating the size of the female sex worker population in three cities in Cote d'Ivoire and in Kisumu, western Kenya.** *Trop Med Int Health* 2010, **15:**1537-1543.

45. Thomsen SC, Gallo MF, Ombidi W, Omungo Z, Janowitz B, Hawken M, Tucker H, Wong EL, Hobbs MM: **Randomised controlled trial on whether advance knowledge of prostate-specific antigen testing improves participant reporting of unprotected sex.** *Sex Transm Infect* 2007, **83:**419-420.

46. Gallo MF, Warner L, Bell AJ, Wiener J, Eschenbach DA, Bukusi EA, Sharma A, Njoroge B, Ngugi E, Jamieson DJ: **Assessment of changes in condom use among female sex workers in a prospective cohort study introducing diaphragm use for disease prevention.** *Am J Epidemiol* 2010, **172:**606-612.

47. Bukusi EA, Gallo MF, Sharma A, Njoroge B, Jamieson DJ, Nguti R, Bell AJ, Eschenbach DA: **Adherence to diaphragm use for infection prevention: a prospective study of female sex workers in Kenya.** *Infect Dis Obstet Gynecol* 2009, **2009:**420196.

48. Luchters S, Chersich MF, Jao I, Schroth A, Chidagaya S, Mandaliya K, Temmerman M: **Acceptability of the diaphragm in Mombasa Kenya: a 6-month prospective study.** *Eur J Contracept Reprod Health Care* 2007, **12:**345-353.

49. Njoroge B, Gallo MF, Sharma A, Bukusi EA, Nguti R, Bell AJ, Jamieson DJ, Williams D, Eschenbach DA: **Diaphragm for STI and HIV prevention: is it a safe method for women at high risk?** *Sex Transm Dis* 2010, **37:**382-385.

50. Masese L, McClelland RS, Gitau R, Wanje G, Shafi J, Kashonga F, Ndinya-Achola JO, Lester R, Richardson BA, Kurth A: **A pilot study of the feasibility of a vaginal washing cessation intervention among Kenyan female sex workers.** *Sex Transm Infect* 2013, **89:**217-222.

51. McClelland L, Wanje G, Kashonga F, Kibe L, McClelland RS, Kiarie J, Mandaliya K, Peshu N, Kurth A: **Understanding the context of HIV risk behavior among HIV-positive and HIV-negative female sex workers and male bar clients following antiretroviral therapy rollout in Mombasa, Kenya.** *AIDS Educ Prev* 2011, **23:**299-312.

52. McClelland RS, Graham SM, Richardson BA, Peshu N, Masese LN, Wanje GH, Mandaliya KN, Kurth AE, Jaoko W, Ndinya-Achola JO: **Treatment with antiretroviral therapy is not associated with increased sexual risk behavior in Kenyan female sex workers.** *AIDS* 2010, **24:**891-897.

53. McClelland RS, Richardson BA, Hassan WM, Chohan V, Lavreys L, Mandaliya K, Kiarie J, Jaoko W, Ndinya-Achola JO, Baeten JM, et al: **Improvement of vaginal health for Kenyan women at risk for acquisition of human immunodeficiency virus type 1: results of a randomized trial.** *J Infect Dis* 2008, **197:**1361-1368.

54. Richardson BA, Lavreys L, Martin HL, Jr., Stevens CE, Ngugi E, Mandaliya K, Bwayo J, Ndinya-Achola J, Kreiss JK: **Evaluation of a low-dose nonoxynol-9 gel for the prevention of sexually transmitted diseases: a randomized clinical trial.** *Sex Transm Dis* 2001, **28:**394-400.

55. Martin HL, Jr., Jackson DJ, Mandaliya K, Bwayo J, Rakwar JP, Nyange P, Moses S, Ndinya-Achola JO, Holmes K, Plummer F, et al.: **Preparation for AIDS vaccine evaluation in Mombasa, Kenya: establishment of seronegative cohorts of commercial sex workers and trucking company employees.** *AIDS Res Hum Retroviruses* 1994, **10 Suppl 2:**S235-237.

56. Baeten JM, Hassan WM, Chohan V, Richardson BA, Mandaliya K, Ndinya-Achola JO, Jaoko W, McClelland RS: **Prospective study of correlates of vaginal Lactobacillus colonisation among high-risk HIV-1 seronegative women.** *Sex Transm Infect* 2009, **85:**348-353.

57. Baeten JM, Richardson BA, Martin HL, Jr., Nyange PM, Lavreys L, Ngugi EN, Mandaliya K, Ndinya-Achola JO, Bwayo JJ, Kreiss JK: **Trends in HIV-1 incidence in a cohort of prostitutes in Kenya: implications for HIV-1 vaccine efficacy trials.** *J Acquir Immune Defic Syndr* 2000, **24:**458-464.

58. Chohan V, Baeten JM, Benki S, Graham SM, Lavreys L, Mandaliya K, Ndinya-Achola JO, Jaoko W, Overbaugh J, McClelland RS: **A prospective study of risk factors for herpes simplex virus type 2 acquisition among high-risk HIV-1 seronegative women in Kenya.** *Sex Transm Infect* 2009, **85:**489-492.

59. Martin HL, Jr., Nyange PM, Richardson BA, Lavreys L, Mandaliya K, Jackson DJ, Ndinya-Achola JO, Kreiss J: **Hormonal contraception, sexually transmitted diseases, and risk of heterosexual transmission of human immunodeficiency virus type 1.** *J Infect Dis* 1998, **178:**1053-1059.

60. Smith DJ, Wakasiaka S, Hoang TD, Bwayo JJ, Del Rio C, Priddy FH: **An evaluation of intravaginal rings as a potential HIV prevention device in urban Kenya: behaviors and attitudes that might influence uptake within a high-risk population.** *J Womens Health (Larchmt)* 2008, **17:**1025-1034.

61. Bandewar SV, Kimani J, Lavery JV: **The origins of a research community in the Majengo Observational Cohort Study, Nairobi, Kenya.** *BMC Public Health* 2010, **10:**630.

62. Van der Elst EM, Mbogua J, Operario D, Mutua G, Kuo C, Mugo P, Kanungi J, Singh S, Haberer J, Priddy F, Sanders EJ: **High Acceptability of HIV Pre-exposure Prophylaxis but Challenges in Adherence and Use: Qualitative Insights from a Phase I Trial of Intermittent and Daily PrEP in At-Risk Populations in Kenya.** *AIDS Behav* 2013, **17:**2162-2172.

63. Kreiss J, Ngugi E, Holmes K, Ndinya-Achola J, Waiyaki P, Roberts PL, Ruminjo I, Sajabi R, Kimata J, Fleming TR, et al.: **Efficacy of nonoxynol 9 contraceptive sponge use in preventing heterosexual acquisition of HIV in Nairobi prostitutes.** *JAMA* 1992, **268:**477-482.

64. Fonck K, Kaul R, Kimani J, Keli F, MacDonald KS, Ronald AR, Plummer FA, Kirui P, Bwayo JJ, Ngugi EN, et al: **A randomized, placebo-controlled trial of monthly azithromycin prophylaxis to prevent sexually transmitted infections and HIV-1 in Kenyan sex workers: study design and baseline findings.** *Int J STD AIDS* 2000, **11:**804-811.

65. Kaul R, Kimani J, Nagelkerke NJ, Fonck K, Ngugi EN, Keli F, MacDonald KS, Maclean IW, Bwayo JJ, Temmerman M, et al: **Monthly antibiotic chemoprophylaxis and incidence of sexually transmitted infections and HIV-1 infection in Kenyan sex workers: a randomized controlled trial.** *JAMA* 2004, **291:**2555-2562.

66. Yadav G, Saskin R, Ngugi E, Kimani J, Keli F, Fonck K, Macdonald KS, Bwayo JJ, Temmerman M, Moses S, Kaul R: **Associations of sexual risk taking among Kenyan female sex workers after enrollment in an HIV-1 prevention trial.** *J Acquir Immune Defic Syndr* 2005, **38:**329-334.

67. Kaul R, Nagelkerke NJ, Kimani J, Ngugi E, Bwayo JJ, Macdonald KS, Rebbaprgada A, Fonck K, Temmerman M, Ronald AR, Moses S: **Prevalent herpes simplex virus type 2 infection is associated with altered vaginal flora and an increased susceptibility to multiple sexually transmitted infections.** *J Infect Dis* 2007, **196:**1692-1697.

68. Ngugi EN, Chakkalackal M, Sharma A, Bukusi E, Njoroge B, Kimani J, MacDonald KS, Bwayo JJ, Cohen CR, Moses S, Kaul R: **Sustained changes in sexual behavior by female sex workers after completion of a randomized HIV prevention trial.** *J Acquir Immune Defic Syndr* 2007, **45:**588-594.

69. Ngugi EN, Wilson D, Sebstad J, Plummer FA, Moses S: **Focused peer-mediated educational programs among female sex workers to reduce sexually transmitted disease and human immunodeficiency virus transmission in Kenya and Zimbabwe.** *J Infect Dis* 1996, **174 Suppl 2:**S240-247.

70. Odek WO, Busza J, Morris CN, Cleland J, Ngugi EN, Ferguson AG: **Effects of micro-enterprise services on HIV risk behaviour among female sex workers in Kenya's urban slums.** *AIDS Behav* 2009, **13:**449-461.

71. Moses S, Plummer FA, Ngugi EN, Nagelkerke NJ, Anzala AO, Ndinya-Achola JO: **Controlling HIV in Africa: effectiveness and cost of an intervention in a high-frequency STD transmitter core group.** *AIDS* 1991, **5:**407-411.

72. Ngugi EN, Plummer FA, Simonsen JN, Cameron DW, Bosire M, Waiyaki P, Ronald AR, Ndinya-Achola JO: **Prevention of transmission of human immunodeficiency virus in Africa: effectiveness of condom promotion and health education among prostitutes.** *Lancet* 1988, **2:**887-890.

73. Ojoo J, Paul J, Batchelor B, Amir M, Kimari J, Mwachari C, Bwayo J, Plummer F, Gachihi G, Waiyaki P, Gilks C: **Bacteriuria in a cohort of predominantly HIV-1 seropositive female commercial sex workers in Nairobi, Kenya.** *J Infect* 1996, **33:**33-37.

74. Witte K, Cameron KA, Lapinski MK, Nzyuko S: **A theoretically based evaluation of HIV/AIDS prevention campaigns along the trans-Africa highway in Kenya.** *J Health Commun* 1998, **3:**345-363.

75. Behets F, Turner AN, Van Damme K, Rabenja NL, Ravelomanana N, Zeller K, Rasolofomanana JR: **Acceptability and feasibility of continuous diaphragm use among sex workers in Madagascar.** *Sex Transm Infect* 2005, **81:**472-476.

76. Behets FM, Rasolofomanana JR, Van Damme K, Vaovola G, Andriamiadana J, Ranaivo A, McClamroch K, Dallabetta G, Van Dam J, Rasamilalao D, Rasamindra A: **Evidence-based treatment guidelines for sexually transmitted infections developed with and for female sex workers.** *Trop Med Int Health* 2003, **8:**251-258.

77. Feldblum PJ, Hatzell T, Van Damme K, Nasution M, Rasamindrakotroka A, Grey TW: **Results of a randomised trial of male condom promotion among Madagascar sex workers.** *Sex Transm Infect* 2005, **81:**166-173.

78. Hoke TH, Feldblum PJ, Van Damme K, Nasution MD, Grey TW, Wong EL, Ralimamonjy L, Raharimalala L, Rasamindrakotroka A: **Temporal trends in sexually transmitted infection prevalence and condom use following introduction of the female condom to Madagascar sex workers.** *Int J STD AIDS* 2007, **18:**461-466.

79. Pettifor AE, Turner AN, Van Damme K, Hatzell-Hoke T, Rasamindrakotroka A, Nasution MD, Behets F: **Increased risk of chlamydial and gonococcal infection in adolescent sex workers in Madagascar.** *Sex Transm Dis* 2007, **34:**475-478.

80. Hoke TH, Feldblum PJ, Damme KV, Nasution MD, Grey TW, Wong EL, Ralimamonjy L, Raharimalala L, Rasamindrakotroka A: **Randomised controlled trial of alternative male and female condom promotion strategies targeting sex workers in Madagascar.** *Sex Transm Infect* 2007, **83:**448-453.

81. Yotebieng M, Turner AN, Hoke TH, Van Damme K, Rasolofomanana JR, Behets F: **Effect of consistent condom use on 6-month prevalence of bacterial vaginosis varies by baseline BV status.** *Trop Med Int Health* 2009, **14:**480-486.

82. McClamroch KJ, Kaufman JS, Behets FM: **A formal decision analysis identifies an optimal treatment strategy in a resource-poor setting.** *J Clin Epidemiol* 2008, **61:**776-787.

83. Smith JS, Van Damme K, Randrianjafisamindrakotroka N, Ting J, Rabozakandraina T, Randrianasolo BS, Raharinivo M, Zanasaotra S, Hobbs M, Rinas A, et al: **Human papillomavirus and cervical neoplasia among female sex workers in Madagascar.** *Int J Gynecol Cancer* 2010, **20:**1593-1596.

84. Pettifor A, Turner AN, Swezey T, Khan M, Raharinivo MS, Randrianasolo B, Penman-Aguilar A, Van Damme K, Jamieson DJ, Behets F: **Perceived control over condom use among sex workers in Madagascar: a cohort study.** *BMC Womens Health* 2010, **10:**4.

85. Penman-Aguilar A, Legardy-Williams J, Turner AN, Rabozakandriana TO, Williams D, Razafindravoavy S, Behets F, Van Damme K, Jamieson DJ: **Effect of treatment assignment on intravaginal cleansing in a randomized study of the diaphragm with candidate microbicide.** *J Womens Health (Larchmt)* 2011, **20:**187-195.

86. Feldblum PJ, Nasution MD, Hoke TH, Van Damme K, Turner AN, Gmach R, Wong EL, Behets F: **Pregnancy among sex workers participating in a condom intervention trial highlights the need for dual protection.** *Contraception* 2007, **76:**105-110.

87. McClamroch K, Behets F, Van Damme K, Rabenja LN, Myers E: **Cost-effectiveness of treatment strategies for cervical infection among women at high risk in Madagascar.** *Sex Transm Dis* 2007, **34:**631-637.

88. Zachariah R, Harries AD, Buhendwa L, Spielman MP, Chantulo A, Bakali E: **Acceptability and technical problems of the female condom amongst commercial sex workers in a rural district of Malawi.** *Trop Doct* 2003, **33:**220-224.

89. Zachariah R, Spielmann MP, Harries AD, Nkhoma W, Chantulo A, Arendt V: **Sexually transmitted infections and sexual behaviour among commercial sex workers in a rural district of Malawi.** *Int J STD AIDS* 2003, **14:**185-188.

90. Walden VM, Mwangulube K, Makhumula-Nkhoma P: **Measuring the impact of a behaviour change intervention for commercial sex workers and their potential clients in Malawi.** *Health Educ Res* 1999, **14:**545-554.

91. Kalanda B: **Empowering young sex workers for safer sex in Dowa and Lilongwe Districts of Malawi.** *Malawi Med J* 2010, **22:**10-11.

92. Lafort Y, Geelhoed D, Cumba L, Lazaro CD, Delva W, Luchters S, Temmerman M: **Reproductive health services for populations at high risk of HIV: Performance of a night clinic in Tete province, Mozambique.** *BMC Health Serv Res* 2010, **10:**144.

93. Fitzgerald-Husek A, Martiniuk AL, Hinchcliff R, Aochamus CE, Lee RB: **"I do what I have to do to survive": an investigation into the perceptions, experiences and economic considerations of women engaged in sex work in Northern Namibia.** *BMC Womens Health* 2011, **11:**35.

94. UNAIDS: **Sex Work and HIV in Namibia: Review of the literature and current programmes.** 2011.

95. Forbi JC, Entonu PE, Mwangi LO, Agwale SM: **Estimates of human immunodeficiency virus incidence among female sex workers in north central Nigeria: implications for HIV clinical trials.** *Trans R Soc Trop Med Hyg* 2011, **105:**655-660.

96. Esu-Williams E, Philips AL, Githens W: **AIDS prevention: a guide for working with commercial sex workers - experiences from Calabar, Nigeria.** AIDSTECH/Family Health International.

97. Imade G, Sagay A, Egah D, Onwuliri V, Grigg M, Egbodo C, Thacher T, Potts M, Short R: **Prevalence of HIV and other sexually transmissible infections in relation to lemon or lime juice douching among female sex workers in Jos, Nigeria.** *Sex Health* 2008, **5:**55-60.

98. Braunstein SL, Ingabire CM, Geubbels E, Vyankandondera J, Umulisa MM, Gahiro E, Uwineza M, Tuijn CJ, Nash D, van de Wijgert JH: **High burden of prevalent and recently acquired HIV among female sex workers and female HIV voluntary testing center clients in Kigali, Rwanda.** *PLoS One* 2011, **6:**e24321.

99. Braunstein SL, Ingabire CM, Kestelyn E, Uwizera AU, Mwamarangwe L, Ntirushwa J, Nash D, Veldhuijzen NJ, Nel A, Vyankandondera J, van de Wijgert JH: **High human immunodeficiency virus incidence in a cohort of Rwandan female sex workers.** *Sex Transm Dis* 2011, **38:**385-394.

100. Braunstein SL, Nash D, Kim AA, Ford K, Mwambarangwe L, Ingabire CM, Vyankandondera J, van de Wijgert JH: **Dual testing algorithm of BED-CEIA and AxSYM Avidity Index assays performs best in identifying recent HIV infection in a sample of Rwandan sex workers.** *PLoS One* 2011, **6:**e18402.

101. Braunstein SL, Umulisa MM, Veldhuijzen NJ, Kestelyn E, Ingabire CM, Nyinawabega J, van de Wijgert JH, Nash D: **HIV diagnosis, linkage to HIV care, and HIV risk behaviors among newly diagnosed HIV-positive female sex workers in Kigali, Rwanda.** *J Acquir Immune Defic Syndr* 2011, **57:**e70-76.

102. Meda N, Ndoye I, M'Boup S, Wade A, Ndiaye S, Niang C, Sarr F, Diop I, Carael M: **Low and stable HIV infection rates in Senegal: natural course of the epidemic or evidence for success of prevention?** *Aids* 1999, **13:**1397-1405.

103. Larsen MM, Sartie MT, Musa T, Casey SE, Tommy J, Saldinger M: **Changes in HIV/AIDS/STI knowledge, attitudes and practices among commercial sex workers and military forces in Port Loko, Sierra Leone.** *Disasters* 2004, **28:**239-254.

104. Pettifor AE, Beksinska ME, Rees HV, Mqoqi N, Dickson-Tetteh KE: **The acceptability of reuse of the female condom among urban South African women.** *J Urban Health* 2001, **78:**647-657.

105. Richter M, Yarrow J, Delany-Moretlwe S: **The women-at-risk project: providing tailored health care to sex workers in inner-city Johannesburg, South Africa.** 2008.

106. Vickerman P, Terris-Prestholt F, Delany S, Kumaranayake L, Rees H, Watts C: **Are targeted HIV prevention activities cost-effective in high prevalence settings? Results from a sexually transmitted infection treatment project for sex workers in Johannesburg, South Africa.** *Sex Transm Dis* 2006, **33:**S122-132.

107. Stadler J, Delany S: **The 'healthy brothel': the context of clinical services for sex workers in Hillbrow, South Africa.** *Cult Health Sex* 2006, **8:**451-464.

108. Dunkle KL, Beksinska ME, Rees VH, Ballard RC, Htun Y, Wilson ML: **Risk factors for HIV infection among sex workers in Johannesburg, South Africa.** *Int J STD AIDS* 2005, **16:**256-261.

109. Pauw I, Brener L: **'You are just whores - you can't be raped': barriers to safe sex practices among women street workers in Cape Town.** *Culture, Health & Sexuality* 2003, **5:**465-481.

110. Williams BG, Taljaard D, Campbell CM, Gouws E, Ndhlovu L, Van Dam J, Carael M, Auvert B: **Changing patterns of knowledge, reported behaviour and sexually transmitted infections in a South African gold mining community.** *AIDS* 2003, **17:**2099-2107.

111. Connolly CA, Ramjee G, Sturm AW, Abdool Karim SS: **Incidence of Sexually Transmitted Infections among HIV-positive sex workers in KwaZulu-Natal, South Africa.** *Sex Transm Dis* 2002, **29:**721-724.

112. Ramjee G, Gouws E: **Prevalence of HIV among truck drivers visiting sex workers in KwaZulu-Natal, South Africa.** *Sex Transm Dis* 2002, **29:**44-49.

113. Ramjee G, Williams B, Gouws E, Van Dyck E, De Deken B, Karim SA: **The impact of incident and prevalent herpes simplex virus-2 infection on the incidence of HIV-1 infection among commercial sex workers in South Africa.** *J Acquir Immune Defic Syndr* 2005, **39:**333-339.

114. Ramjee G, Karim SS, Sturm AW: **Sexually transmitted infections among sex workers in KwaZulu-Natal, South Africa.** *Sex Transm Dis* 1998, **25:**346-349.

115. Karim QA, Karim SS, Soldan K, Zondi M: **Reducing the risk of HIV infection among South African sex workers: socioeconomic and gender barriers.** *Am J Public Health* 1995, **85:**1521-1525.

116. Auvert B, Marais D, Lissouba P, Zarca K, Ramjee G, Williamson AL: **High-risk human papillomavirus is associated with HIV acquisition among South African female sex workers.** *Infect Dis Obstet Gynecol* 2011, **2011:**692012.

117. Bures R, Morris L, Williamson C, Ramjee G, Deers M, Fiscus SA, Abdool-Karim S, Montefiori DC: **Regional clustering of shared neutralization determinants on primary isolates of clade C human immunodeficiency virus type 1 from South Africa.** *J Virol* 2002, **76:**2233-2244.

118. Rustomjee R, Abdool Karim Q, Abdool Karim SS, Laga M, Stein Z: **Phase 1 trial of nonoxynol-9 film among sex workers in South Africa.** *AIDS* 1999, **13:**1511-1515.

119. Vandebosch A, Goetghebeur E, Ramjee G, Alary M, Ettiegne-Traore V, Chandeying V, Van Damme L: **Acceptability of COL-1492, a vaginal gel, among sex workers in one Asian and three African cities.** *Sex Transm Infect* 2004, **80:**241-243.

120. Steen R, Dallabetta G: **The use of epidemiologic mass treatment and syndrome management for sexually transmitted disease control.** *Sex Transm Dis* 1999, **26:**S12-20; discussion S21-12.

121. Steen R, Vuylsteke B, DeCoito T, Ralepeli S, Fehler G, Conley J, Bruckers L, Dallabetta G, Ballard R: **Evidence of declining STD prevalence in a South African mining community following a core-group intervention.** *Sex Transm Dis* 2000, **27:**1-8.

122. Marseille E, Kahn JG, Billinghurst K, Saba J: **Cost-effectiveness of the female condom in preventing HIV and STDs in commercial sex workers in rural South Africa.** *Soc Sci Med* 2001, **52:**135-148.

123. Wechsberg WM, Luseno WK, Lam WK: **Violence against substance-abusing South African sex workers: intersection with culture and HIV risk.** *AIDS Care* 2005, **17 Suppl 1:**S55-64.

124. Wechsberg WM, Luseno WK, Lam WK, Parry CD, Morojele NK: **Substance use, sexual risk, and violence: HIV prevention intervention with sex workers in Pretoria.** *AIDS Behav* 2006, **10:**131-137.

125. Family Health International: **Corridors of Hope in Southern Africa: HIV Prevention Needs and Opportunities in Four Border Towns.** 2005.

126. Agha S, Chulu Nchima M: **Life-circumstances, working conditions and HIV risk among street and nightclub-based sex workers in Lusaka, Zambia.** *Cult Health Sex* 2004, **6:**283-299.

127. Agha S, Karlyn A, Meekers D: **The promotion of condom use in non-regular sexual partnerships in urban Mozambique.** *Health Policy Plan* 2001, **16:**144-151.

128. Laukamm-Josten U, Mwizarubi BK, Outwater A, Mwaijonga CL, Valadez JJ, Nyamwaya D, Swai R, Saidel T, Nyamuryekung'e K: **Preventing HIV infection through peer education and condom promotion among truck drivers and their sexual partners in Tanzania, 1990-1993.** *AIDS Care* 2000, **12:**27-40.

129. Pepin J, Morgan G, Dunn D, Gevao S, Mendy M, Gaye I, Scollen N, Tedder R, Whittle H: **HIV-2-induced immunosuppression among asymptomatic West African prostitutes: evidence that HIV-2 is pathogenic, but less so than HIV-1.** *AIDS* 1991, **5:**1165-1172.

130. Pepin J, Dunn D, Gaye I, Alonso P, Egboga A, Tedder R, Piot P, Berry N, Schellenberg D, Whittle H, et al.: **HIV-2 infection among prostitutes working in The Gambia: association with serological evidence of genital ulcer diseases and with generalized lymphadenopathy.** *AIDS* 1991, **5:**69-75.

131. Pickering H, Quigley M, Hayes RJ, Todd J, Wilkins A: **Determinants of condom use in 24,000 prostitute/client contacts in The Gambia.** *AIDS* 1993, **7:**1093-1098.

132. Pickering H, Quigley M, Pepin J, Todd J, Wilkins A: **The effects of post-test counselling on condom use among prostitutes in The Gambia.** *AIDS* 1993, **7:**271-273.

133. Hawkes S, West B, Wilson S, Whittle H, Mabey D: **Asymptomatic carriage of Haemophilus ducreyi confirmed by the polymerase chain reaction.** *Genitourinary medicine* 1995, **71:**224-227.

134. Sobela F, Pepin J, Gbeleou S, Banla AK, Pitche VP, Adom W, Sodji D, Frost E, Deslandes S, Labbe AC: **A tale of two countries: HIV among core groups in Togo.** *J Acquir Immune Defic Syndr* 2009, **51:**216-223.

135. Vandepitte J, Bukenya J, Weiss HA, Nakubulwa S, Francis SC, Hughes P, Hayes R, Grosskurth H: **HIV and other sexually transmitted infections in a cohort of women involved in high-risk sexual behavior in Kampala, Uganda.** *Sex Transm Dis* 2011, **38:**316-323.

136. The World Bank: **HIV/AIDS Project For Abidjan/Lagos Transport Corridor. Report No: ICR0000615.** 2008.

137. Morin D, Godin G, Alary M, Sawadogo MR, Bernier M, Khonde N, Kintin F, Kone A, N'Dour M, Pepin J, et al: **Satisfaction with health services for STIs, HIV, AIDS among a high-risk population in West Africa.** *AIDS Care* 2008, **20:**388-394.

138. Jain S, Greene M, Douglas Z, Betron M, Fritz K: **Risky Business Made Safer. Corridors of Hope: An HIV Prevention Program in Zambian Border and Transit Towns. USAID.** 2008.

139. Wilson D, Sibanda B, Mboyi L, Msimanga S, Dube G: **A pilot study for an HIV prevention programme among commercial sex workers in Bulawayo, Zimbabwe.** *Soc Sci Med* 1990, **31:**609-618.

140. Ray S, van De Wijgert J, Mason P, Ndowa F, Maposhere C: **Constraints faced by sex workers in use of female and male condoms for safer sex in urban zimbabwe.** *J Urban Health* 2001, **78:**581-592.

141. Mason PR, Gregson S, Gwanzura L, Cappuccinelli P, Rapelli P, Fiori PL: **Enzyme immunoassay for urogenital trichomoniasis as a marker of unsafe sexual behaviour.** *Epidemiol Infect* 2001, **126:**103-109.

142. Cowan FM, Hargrove JW, Langhaug LF, Jaffar S, Mhuriyengwe L, Swarthout TD, Peeling R, Latif A, Bassett MT, Brown DW, et al: **The appropriateness of core group interventions using presumptive periodic treatment among rural Zimbabwean women who exchange sex for gifts or money.** *J Acquir Immune Defic Syndr* 2005, **38:**202-207.

143. Cowan FF, Pascoe SJ, Barlow KL, Langhaug LF, Jaffar S, Hargrove JW, Robinson NJ, Latif AS, Bassett MT, Wilson D, et al: **Association of genital shedding of herpes simplex virus type 2 and HIV-1 among sex workers in rural Zimbabwe.** *AIDS* 2006, **20:**261-267.

144. Cowan FM, Pascoe SJ, Barlow KL, Langhaug LF, Jaffar S, Hargrove JW, Robinson NJ, Bassett MT, Wilson D, Brown DW, Hayes RJ: **A randomised placebo-controlled trial to explore the effect of suppressive therapy with acyclovir on genital shedding of HIV-1 and herpes simplex virus type 2 among Zimbabwean sex workers.** *Sex Transm Infect* 2008, **84:**548-553.

145. Chipfakacha V: **Prevention of sexually transmitted disease. The Shurugwi sex-workers project.** *S Afr Med J* 1993, **83:**40-41.

146. Leonard L, Ndiaye I, Kapadia A, Eisen G, Diop O, Mboup S, Kanki P: **HIV prevention among male clients of female sex workers in Kaolack, Senegal: results of a peer education program.** *AIDS Educ Prev* 2000, **12:**21-37.

147. Wang C, Hawes SE, Gaye A, Sow PS, Ndoye I, Manhart LE, Wald A, Critchlow CW, Kiviat NB: **HIV prevalence, previous HIV testing, and condom use with clients and regular partners among Senegalese commercial sex workers.** *Sex Transm Infect* 2007, **83:**534-540.

148. Laurent C, Seck K, Coumba N, Kane T, Samb N, Wade A, Liegeois F, Mboup S, Ndoye I, Delaporte E: **Prevalence of HIV and other sexually transmitted infections, and risk behaviours in unregistered sex workers in Dakar, Senegal.** *AIDS* 2003, **17:**1811-1816.

149. Nyamuryekung'e K, Laukamm-Josten U, Vuylsteke B, Mbuya C, Hamelmann C, Outwater A, Steen R, Ocheng D, Msauka A, Dallabetta G: **STD services for women at truck stop in Tanzania: evaluation of acceptable approaches.** *East Afr Med J* 1997, **74:**343-347.
